# Recent Progress in Nanophotonics for Green Energy, Medicine, Healthcare, and Optical Computing Applications

**DOI:** 10.3390/ma19081660

**Published:** 2026-04-21

**Authors:** Osama M. Halawa, Esraa Ahmed, Malk M. Abdelrazek, Yasser M. Nagy, Omar A. M. Abdelraouf

**Affiliations:** 1 Mechanical Engineering Department, Faculty of Engineering at El-Mataria, Helwan University, Cairo 11718, Egypt; osama20190113@m-eng.helwan.edu.eg; 2 Laser Institute for Research and Applications LIRA, Beni-Suef University, Beni-Suef 62511, Egypt; esr3ahmed@lira.bsu.edu.eg; 3 Beni-Suef Students Research Unit, Faculty of Medicine, Beni-Suef University, Beni-Suef 62511, Egypt; malaakmohamed325_sd@med.bsu.edu.eg (M.M.A.); yassermohamed396_sd@med.bsu.edu.eg (Y.M.N.); 4 Institute of Materials Research and Engineering, Agency for Science, Technology, and Research (A*STAR), 2 Fusionopolis Way, #08-03, Innovis, Singapore 138634, Singapore

**Keywords:** nanophotonics, MedTech, healthcare, solar energy, optical computing, medicine

## Abstract

Nanophotonics, an interdisciplinary field merging nanotechnology and photonics, has enabled transformative advancements across diverse sectors, including green energy, biomedicine, and optical computing. This review comprehensively examines recent progress in nanophotonic principles and applications, highlighting key innovations in material design, device engineering, and system integration. In renewable energy, nanophotonics allows the use of light-trapping nanostructures and spectral control in perovskite solar cells, concentrating solar power systems, and thermophotovoltaics. This has significantly enhanced solar conversion efficiencies, approaching theoretical limits. In biosensing, nanophotonic platforms achieve unprecedented sensitivity in detecting biomolecules, pathogens, and pollutants, enabling real-time diagnostics and environmental monitoring. Medical applications leverage tailored light–matter interactions for precision photothermal therapy, image-guided surgery, and early disease detection. Furthermore, nanophotonics underpins next-generation optical neural networks and neuromorphic computing, offering ultrafast, energy-efficient alternatives to von Neumann architectures. Despite rapid growth, challenges in scalability, fabrication costs, and material stability persist. Future advancements will rely on novel materials, AI-driven design optimization, and multidisciplinary approaches to enable scalable, low-cost deployment. This review summarizes recent progress and highlights future trends, including novel material systems, multidisciplinary approaches, and enhanced computational capabilities, paving the way for transformative applications in this rapidly evolving field.

## 1. Introduction

### 1.1. Overview of Nanophotonics and Its Significance

Light, the fundamental carrier of information and energy, has been harnessed by humanity for millennia through traditional optics, employing lenses, mirrors, and prisms crafted from bulk materials. While foundational to scientific progress, these macroscopic components face inherent limitations as technology advances toward miniaturization, enhanced efficiency, and novel functionalities. Refractive optics fundamentally constrains resolution and device size, and the physical scale hinders integration into compact systems like semiconductor chips. Refractive optics has weak light–matter interactions in bulk media and requires high power or long path lengths for significant nonlinear effects or efficient sensing. Nanophotonics has emerged as a transformative solution to these challenges. This vibrant, interdisciplinary field, operating at the intersection of optics, photonics, materials science, nanotechnology, and quantum physics, focuses on manipulating light using structures and devices with critical features on the scale of, or smaller than, the wavelength of light itself, typically tens to hundreds of nanometers [1,2,3].

By engineering materials and geometries at this subwavelength scale, nanophotonics circumvents the diffraction limit and enables unprecedented control over light propagation, localization, emission, and absorption [4,5]. This mastery unlocks phenomena impossible or highly inefficient in bulk optics: confining light to volumes far smaller than its wavelength, sculpting optical wavefronts with ultra-thin components, and dramatically enhancing light–matter interactions. These capabilities represent not merely incremental improvements but a paradigm shift, paving the way for revolutionary devices and applications across diverse sectors, including sustainable energy, life-saving healthcare, ultrafast computing, and quantum technologies [6,7,8].

**Figure 1:**
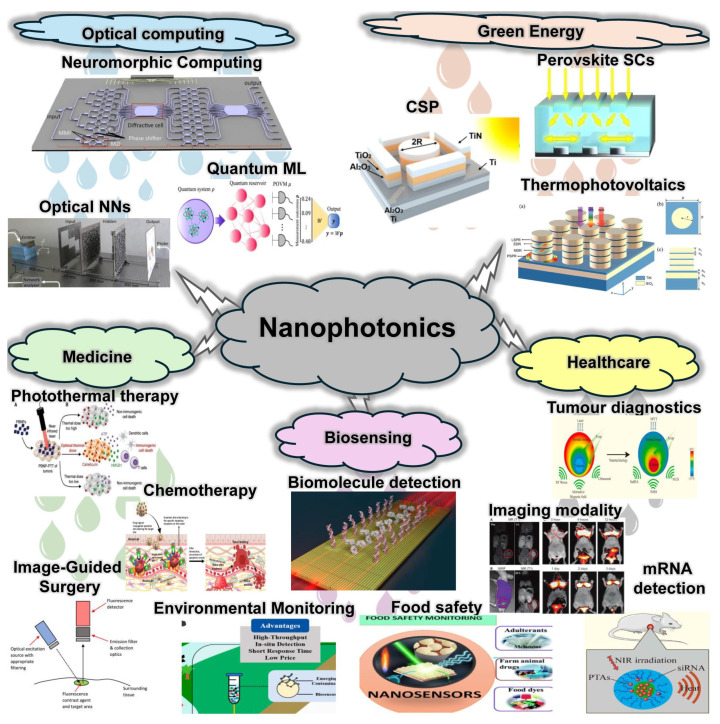
Nanophotonic application clouds. Reprinted with permission from [25,26,27,28,29,30,31,32,33,34,35,36].

The profound advantages of nanophotonics stem directly from its ability to transcend classical optical constraints and exploit unique nanoscale physics. Critically, it breaks the diffraction limit; structures like plasmonic nanoantennas or high-index dielectric resonators concentrate optical energy into “hot spots” with dimensions significantly smaller than half the wavelength. This enables super-resolution imaging, ultra-compact components, and molecular-level probing. Furthermore, it facilitates ultra-compact integration. Replacing bulky lenses and mirrors with sub-micron planar devices like metasurfaces allows complex optical systems to be miniaturized and integrated directly onto semiconductor chips, enabling photonic integrated circuits (PICs) and lab-on-a-chip platforms. Perhaps most significantly, nanophotonics dramatically enhances light–matter interactions by confining light to extremely small volumes, which increases local electromagnetic field intensity and photon density of states, thereby profoundly boosting absorption in photodetectors and solar cells. This also enhances the emission rate of quantum emitters for brighter sources [9,10,11,12,13], lowers power thresholds for nonlinear optical effects like harmonic generation [14,15,16,17,18,19], and amplifies sensitivity to minute environmental changes for advanced sensing (Figure 1) [20,21,22,23,24]. Additionally, it enables the tailored engineering of optical properties, creating metamaterials with exotic characteristics like negative refraction and metasurfaces with spatially varying responses for complex wavefront shaping, all while consuming orders of magnitude less material than bulk optics, offering benefits in cost, sustainability, and weight.

### 1.2. Fundamental Physics of Nanophotonics

**Figure 2:**
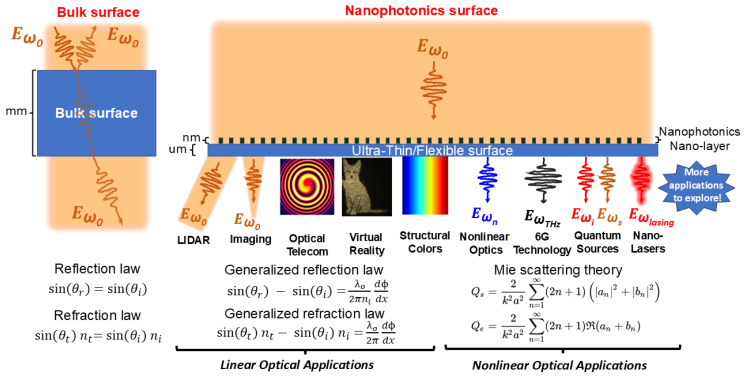
Nanophotonic surfaces (metasurfaces) vs. bulk photonic devices.

The foundation of nanophotonics lies in meticulously designed nanostructures governed by fundamental physical principles. A revolutionary concept is the metasurface, an artificial sheet material, nanometers to a few hundred nanometers thick, composed of dense arrays of subwavelength nanostructures (meta-atoms). By precisely designing the geometry, material, and arrangement of these meta-atoms, local control over the amplitude, phase, and polarization of scattered light is achieved. A cornerstone achievement is the phase-gradient metasurface. By imposing a linear spatial gradient in the phase shift imparted by adjacent meta-atoms, these devices anomalously refract or reflect incident light according to the generalized laws of reflection and refraction. Unlike Snell’s law governing homogeneous bulk interfaces, the generalized laws predict deflection angles dependent on both the refractive index difference and the deliberately introduced phase gradient (Figure 2) [37,38,39,40,41].

(1) $$\sin(\theta_{r})\;-\;\sin(\theta_{i})=\frac{\lambda_{o}}{2\pi n_{i}}\frac{d\phi }{dx}$$

(2) $$\sin(\theta_{t})\;n_{t}-\;\sin(\theta_{i})\;n_{i}=\frac{\lambda_{o}}{2\pi }\frac{d\phi }{dx}$$

where *n_i_* and *n_t_* are the incident and transmitted refractive indices, *θ_i_* and *θ_t_* are the incident and transmitted angles, *λ*_0_ is the free-space wavelength, and $\frac{d\phi }{dx}$ is the phase gradient. This principle enables ultra-thin flat lenses (metalenses), beam steerers, holograms, and polarizers, replacing bulky optics with planar, CMOS-compatible components capable of sophisticated wavefront manipulation. Achieving deep subwavelength light confinement and intense field enhancement relies heavily on resonant phenomena. Mie scattering theory provides the fundamental framework for understanding electromagnetic wave interactions with subwavelength dielectric objects (e.g., silicon and GaAs nanoparticles). High-index dielectrics support Mie resonances, including magnetic dipole (MD, from circular displacement currents), electric dipole (ED, from oscillating polarization), and higher-order multipoles. The governing equations for diploe and quadrupole optical modes are [42,43,44]

(3) $$C_{ED}=\frac{k_{0}^{4}}{6\;\pi \epsilon_{0}^{2}E_{0}^{2}}{\left|p_{car}+\frac{ik_{0}}{c}\left(t+\frac{k_{0}^{2}}{10}\bar{R_{t}^{2}}\right)\right|}^{2}\;$$

(4) $$C_{EQ}=\frac{k_{0}^{6}}{80\;\pi \epsilon_{0}^{2}E_{0}^{2}}{\left|\overset{̿}{Q_{e}}+\frac{ik_{0}}{c}\overset{̿}{Q_{t}}\right|}^{2}$$

(5) $$C_{MD}=\frac{{\eta_{0}^{2}k}_{0}^{4}}{6\;\pi E_{0}^{2}}{\left|m_{car}-k_{0}^{2}\bar{R_{m}^{2}}\right|}^{2}\;$$

(6) $$C_{MQ}=\frac{{\eta_{0}^{2}k}_{0}^{6}}{80\;\pi E_{0}^{2}}{\left|\overset{̿}{Q_{m}}\right|}^{2}$$

where *C_ED_*, *C_MD_*, *C_EQ_*, and *C_MQ_* denote the scattering cross-sections of dipole modes for electric and magnetic fields and those of quadrupolar modes for electric and magnetic fields, respectively. Optical modes arise from displacement currents within the material and offer low optical losses compared to plasmonic metals. Multi-optical mode metasurfaces represent a sophisticated evolution, where nanostructures are engineered to simultaneously support multiple, spectrally distinct Mie resonances. This multimodal capability is pivotal for nonlinear optics, where resonances at both fundamental pump and generated harmonic wavelengths boost interaction strength; specific modal overlaps can enable or enhance processes and facilitate unique phase-matching, while extreme field confinement amplifies nonlinear coefficients. It is equally crucial for quantum optics, where resonant structures enhance the spontaneous emission rate of quantum emitters (the Purcell effect) via tailored modal profiles. Designing a multimode metasurface will enable control emission directionality, polarization, and efficiency, as well as strong light–matter coupling for quantum information processing and nonlinear quantum optics at the single-photon level [45,46,47].

The unique capabilities unlocked by nanophotonic design principles are actively driving transformative solutions across critical global challenges and enabling novel functionalities. In the realm of green energy, nanophotonics significantly enhances efficiency. For photovoltaics, nanostructured surfaces minimize reflection losses, while light-trapping schemes using nanostructures increase the effective optical path length within absorber layers, boosting absorption in thin-film technologies; spectral splitting better matches sunlight to multijunction cell bandgaps, pushing efficiencies closer to theoretical limits with reduced material costs. In photocatalysis, plasmonic nanoparticles act as nanoantennas by concentrating light to generate hot electrons for driving reactions like water splitting, while dielectric nanostructures enhance absorption within semiconductors and improve charge separation, enabling efficient systems for sustainable fuel production and environmental remediation [48].

Biosensing and environmental monitoring benefit tremendously from accurate and non-invasive inspection. Label-free biosensing leverages the exquisite refractive index sensitivity of nanophotonic resonators (plasmonic nanostructures, photonic crystals, and dielectric metasurfaces); the binding of target biomolecules to functionalized surfaces induces detectable spectral shifts, enabling real-time, highly sensitive, multiplexed detection for disease diagnosis and point-of-care testing. Similarly, functionalized nanophotonic sensors provide real-time, highly sensitive, and selective monitoring of pollutants in air and water, facilitated by miniaturized, rugged sensor nodes for widespread environmental surveillance. Healthcare has seen profound impacts in diagnosis and safe, targeted treatment. In advanced imaging, nanophotonics is leveraged to achieve super-resolution microscopy by breaking the diffraction barrier and enhancing contrast in techniques like optical coherence tomography (OCT), while nanophotonic biosensors offer rapid diagnostics. Crucially, safe cancer optothermal therapy utilizes plasmonic nanoparticles (e.g., gold nanorods) engineered to absorb near-infrared light within the “biological window”; injected into tumors and illuminated, they efficiently convert light into localized heat, selectively ablating cancerous cells with minimal damage to healthy tissue, offering a minimally invasive alternative [33].

At present, nanophotonics is powering the next generation of computing. For high-speed interconnects, nanoscale waveguides, modulators, and detectors on silicon chips enable ultrafast, low-power optical communication between processor cores and memory, alleviating the “von Neumann bottleneck.” In neuromorphic computing, nanophotonic components like metasurfaces and programmable photonic circuits perform complex linear operations (matrix multiplications) inherent in artificial neural networks at the speed of light with minimal energy; nonlinear nanophotonic elements implement activation functions, promising revolutionary gains in speed and efficiency for AI and complex data analysis [49].

Nanophotonics stands as a defining technology of the modern era, fundamentally reshaping our mastery over light and its interaction with matter. By overcoming the diffraction barrier through nanoscale engineering, it unlocks unparalleled capabilities: extreme light confinement, precise wavefront control with ultra-thin devices, and massively amplified light–matter interactions. The foundational physics of generalized refraction enabled by phase-gradient metasurfaces, as well as the intricate multi-resonant phenomena governed by Mie theory in dielectric nanostructures, provides a powerful design framework for controlling nonlinear light emission and polarization of light–matter interactions at the nanoscale. The following sections of this review will delve deeper into the role of this technology in boosting green energy efficiency, enabling ultra-sensitive biosensing and environmental monitoring, pioneering safe cancer therapies and efficient healthcare, and laying the groundwork for next-generation optical computing.

## 2. Nanophotonics for Energy Production and Conversion

The quest for efficient and cost-effective solar energy conversion has driven remarkable innovations in nanophotonics over the past decade. Nanophotonics, the study of light behavior at the nanometer scale, has enabled breakthrough technologies that manipulate light–matter interactions at the nanoscale to enhance solar energy harvesting [50,51,52,53,54,55,56,57,58]. This section of the review focuses on three transformative technologies that exemplify the power of nanophotonics in solar energy: perovskite solar cells, concentrating solar power systems, and solar thermophotovoltaics. These technologies represent different approaches to solar energy conversion, each leveraging nanophotonic principles to overcome traditional limitations and achieve unprecedented performance levels. From the rapid efficiency improvements in perovskite solar cells to the enhanced light concentration capabilities of solar power systems, nanophotonics continues to revolutionize how we capture and convert solar energy.

### 2.1. Perovskite Solar Cells

#### 2.1.1. Overview of Perovskite Solar Cell Technology

Perovskite solar cells (PSCs) represent one of the most promising and rapidly advancing photovoltaic technologies of the 21st century. Perovskite solar cells represent photovoltaic devices that utilize perovskite-structured compounds functioning as the primary light-absorbing component within the device architecture [59]. The nomenclature derives from the naturally occurring mineral CaTiO_3_, initially identified in the Ural Mountain region by Gustav Rose during the nineteenth century and subsequently named to honor Russian mineralogist Lev Perovskite’s scientific contributions [60,61,62,63,64,65,66,67,68].

The implementation of nanophotonic designs has demonstrated remarkable success in improving perovskite solar cell efficiencies, with recent advances showing progression from 3.8% to over 25.5% for single-junction devices and exceeding the certified 30% for tandem configurations. Furthermore, nanophotonic structures offer unique advantages for perovskite devices by enabling better integration compatibility than conventional silicon-based approaches while simultaneously providing pathways for enhanced stability and reduced manufacturing complexity.

#### 2.1.2. Recent Developments in Perovskite Solar Cells

Perovskite photovoltaic technology has undergone substantial technological advancement throughout the contemporary research period, characterized by marked improvements in electrical conversion performance, operational durability, architectural configuration designs, and innovative material synthesis methodologies. This section examines the latest developments based on cutting-edge research that has pushed the boundaries of perovskite photovoltaic technology toward commercial viability.

##### Advanced Optical Characterization and Performance Enhancement

Recent studies have highlighted the essential role of accurate electromagnetic property measurement in advancing perovskite photovoltaic device functionality and performance capabilities. The research group led by Widianto conducted a thorough exploration of how spectroscopic ellipsometry techniques can be strategically deployed to improve perovskite-based photovoltaic system performance [69]. The study revealed that spectroscopic ellipsometry instrumentation provides fundamental insights into pivotal electromagnetic characteristics, including dielectric response properties, refractive index variations, and spectral absorption coefficients of perovskite thin-film materials. The study stressed that detailed knowledge of optical property behavior represents an essential precondition for developing enhanced photovoltaic device geometric arrangements and maximizing overall functional performance outcomes.

The study also revealed that spectroscopic ellipsometry enables a systematic evaluation of optoelectronic properties in complex, multilayered perovskite systems. This analytical methodology has demonstrated exceptional utility in examining thermal response phenomena, heat-induced material deterioration mechanisms, and compositional influences that fundamentally govern both the operational performance and environmental durability of photovoltaic cell systems. The capacity to conduct rigorous optical property assessment represents meaningful scientific progress, equipping researchers with essential analytical instruments to refine light-harvesting efficiency and thereby advance electrical conversion performance in perovskite-based solar cells.

Moreover, spectroscopic ellipsometry-based methodologies function as robust analytical tools for comprehensive assessment of perovskite material thin-film characteristics, with emphasis on the development of optical property models, quantitative analysis frameworks, and the determination of fundamental optical parameters. This comprehensive approach to optical characterization has opened new pathways for accelerating the commercialization of perovskite solar cells by providing deeper insights into the fundamental optical processes that govern device performance [69].

##### Breakthrough Achievements in Tandem Solar Cell Technology

The development of tandem solar cell architectures represents one of the most promising approaches for surpassing the efficiency limitations of single-junction devices. Qian et al. reported groundbreaking achievements in four-terminal tandem devices through innovative light utilization optimization strategies [70]. The investigation concentrated on partially transparent perovskite photovoltaic modules and presented an innovative strategy through the integration of tin oxide nanoparticles (SnO_2_ NPs) within the perovskite precursor formulation. Ceramic oxides, binary oxides, and transition metal oxides have enhanced solar cell performance in general [71,72,73].

This creative methodology facilitated the formation of p-type/n-type homojunction structures within the upper absorber layer of large-scale thin-film configurations, thereby substantially enhancing device operational characteristics. The investigation established that this technique strengthens the internal electric field through homojunction formation mechanisms while concurrently improving visible-spectrum photon diffusion patterns throughout the perovskite absorber layer via light-scattering phenomena induced by integrated nanoparticles. The synergistic combination of these effects has demonstrated considerable efficacy in advancing both charge carrier transport performance and optical management strategies.

The experimental results achieved by this research team are particularly noteworthy. The 56.9 cm^2^ semi-transparent perovskite modules attained a validated electrical conversion efficiency of 17.2%, constituting a noteworthy scientific achievement in large-scale photovoltaic device capabilities. Upon mechanical integration with silicon heterojunction photovoltaic cells to establish four-terminal tandem configurations, the combined system reached a power conversion efficiency of 27.2%. This result underscores the considerable promise of stacked architectures in transcending the fundamental efficiency boundaries inherent in single-absorber photovoltaic systems.

The broader implications of this work transcend mere efficiency improvements, as it established innovative conceptual frameworks for systematically advancing perovskite tandem photovoltaic system performance. The investigation identified viable development pathways for forthcoming photovoltaic energy technologies by illustrating how precisely designed nanostructures can concurrently mitigate numerous efficiency-constraining phenomena within perovskite-based devices.

##### Revolutionary Advances in Hole Transport Materials

Progress in charge transport layer engineering has become increasingly essential for realizing high-performance perovskite photovoltaic systems. Recent research by the Nanophotonics Team at Guangxi University has yielded a series of groundbreaking achievements in this area. Their work represents a systematic approach to material innovation that has resulted in some of the highest reported efficiencies for perovskite solar cells.

One of the most significant contributions from this research group is the design and synthesis of a novel hole transport material named V3PACz with in situ crosslinking capability. This breakthrough was achieved by combining an in situ self-polymerization strategy with carbazole phosphonic acid self-assembled molecules [74]. The innovative approach enabled the fabrication of high-quality Poly-V3PACz hole transport layers, which achieved an outstanding power conversion efficiency of 25.21%, along with excellent stability characteristics.

The investigators also made important progress in developing organic small-molecule dopants for active layer modification. They formulated and manufactured two PDI-based n-type molecules with A-D-A configurations, namely, PBDT and PTBDT, substantially improving perovskite film crystallinity, charge extraction efficiency, trap-state passivation, and long-term stability [55]. Incorporating these compounds into devices with a 0.08 cm^2^ active area yielded an impressive power conversion efficiency of 25.94%, along with a 1.18 V open-circuit voltage and 86.37% fill factor. These performance metrics rank among the highest open-circuit voltage and efficiency values reported to date for inverted single-junction perovskite solar cells.

Perhaps most innovatively, the research team pioneered the use of deuteration in material design by developing a novel self-assembling hole transport material, 4PACzd8. Perovskite photovoltaic devices incorporating 4PACzd8 as the charge transport interface demonstrated remarkable electrical conversion performance of 24.87%, combined with superior resistance to ultraviolet radiation-induced degradation [75]. This study demonstrated the significant value of the deuteration strategy in the design of small-molecule hole transport materials, opening new avenues for material engineering in perovskite photovoltaics.

##### Nano-Optical Engineering for Enhanced Performance

The incorporation of nanoscale optical architectural principles has demonstrated substantial effectiveness as a methodology for enhancing the operational performance of layered perovskite–silicon photovoltaic systems [73,74]. Tockhorn and colleagues conducted a detailed investigation of perovskite–silicon stacked photovoltaic cells incorporating periodic nanoscale surface patterning, demonstrating substantial performance benefits while preserving the crystalline quality of liquid-solution-deposited perovskite absorber materials. Their research addressed one of the fundamental challenges in tandem device development: optimizing optical performance while maintaining high-quality film growth.

The study demonstrated that periodic nanotextures provide substantial benefits over conventional planar tandem devices. The nanotextured devices showed a significant reduction in reflection losses while exhibiting reduced sensitivity to deviations from optimum layer thicknesses. This characteristic is particularly important for manufacturing scalability, as it provides greater tolerance to process variations that are inevitable in large-scale production.

One of the most remarkable achievements of this research was the dramatic improvement in fabrication yield. Integration of nanoscale surface patterning facilitated a substantial increase in production efficiency from 50% to 95%, signifying a near-doubling of manufacturing output reliability. This enhancement is crucial for the commercial viability of perovskite tandem technology, as it directly impacts production costs and scalability.

**Figure 3:**
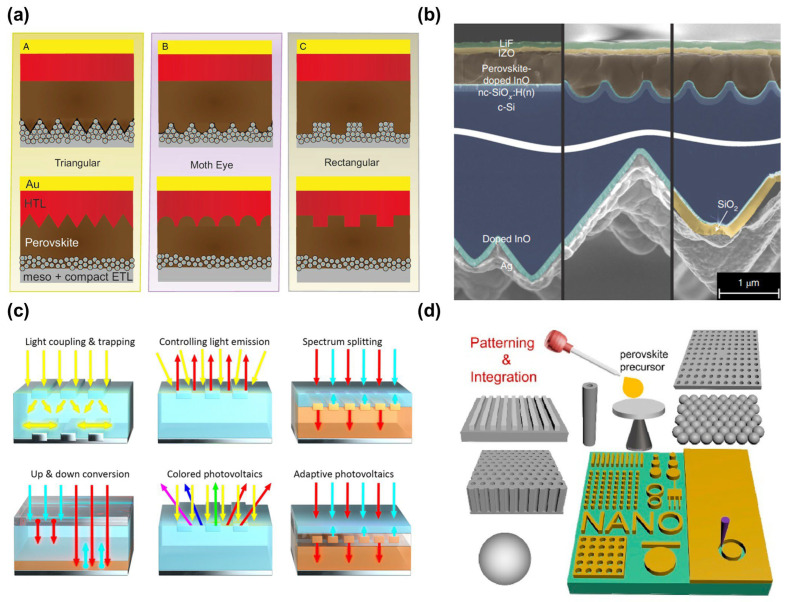
Some of the recent advancements in nanophotonics for improving perovskite solar cell performance. (**a**) Nanostructured designs with triangular interfaces, moth-eye architectures, and rectangular interfaces [68]. (**b**) SEM cross-sections of planar, nanotextured, and nanotextured + RDBL PSTSCs, showing front and rear sides, with c-Si indicating crystalline silicon [76]. (**c**) Light management structures for enhanced photovoltaic efficiency [25]. (**d**) Advanced patterning and integration methodologies for fabricating micro- and nanostructured perovskite materials [77].

The investigation additionally established that nanoscale texturing enhances the electrical and optical characteristics of the perovskite absorber layer, improving the maximum output voltage by 15 millivolts. The research group further engineered a spectrally optimized back-reflecting interface incorporating an insulating intermediate layer that minimized unproductive photon absorption in the near-infrared spectral range. The integration of these technological innovations resulted in independently validated electrical conversion performance of 29.80%, confirming the substantial promise of nanoscale optical engineering methodologies (Figure 3) [76].

##### Advanced Light Management and Photonic Structures

Recent developments in light management techniques have focused on addressing both efficiency and stability challenges simultaneously. Haque et al. introduced innovative photonic structures that combine light trapping with UV protection capabilities. The investigation features a checkered geometric motif incorporating specialized ultraviolet photon transformation properties, exemplifying creative methodological approaches to mitigating numerous efficiency constraints in perovskite photovoltaic devices.

Utilizing integrated computational simulations involving electromagnetic and charge transport phenomena, the study establishes that this photonic design framework improves the electrical current generation and energy conversion performance of thickness-reduced perovskite cells by 25.9% and 28.2%, respectively. These improvements are particularly significant for ultra-thin devices, where optical losses typically limit performance.

The investigation additionally presents a photon-converting protective coating material that transforms ultraviolet radiation into visible-range electromagnetic radiation aligned with photovoltaic cell spectral sensitivity. This methodology tackles fundamental environmental durability constraints in perovskite systems, given established knowledge that ultraviolet exposure induces material decomposition phenomena. The investigation confirms that a minimum of 94% of incident ultraviolet radiation undergoes successful conversion to visible wavelengths, concurrently delivering improvements in efficiency and environmental robustness.

This research constitutes meaningful scientific progress in simultaneously tackling operational efficiency and temporal durability constraints within perovskite photovoltaic technology. Through the integration of photon confinement methodologies with wavelength-converting protective interfaces, the investigation unveils feasible optical design principles for mitigating ultraviolet-induced material deterioration and eliminating unwanted light dissipation in minimal-thickness photovoltaic configurations. This design proves particularly applicable to extraterrestrial deployment under AM0 solar spectral conditions, characterized by substantially elevated ultraviolet flux relative to Earth-based operating environments [78].

##### Nanoscale Material Engineering and Enhancement Strategies

The strategic incorporation of nanoscale materials has emerged as a powerful approach for enhancing perovskite solar cell performance. Aftab and colleagues performed a thorough examination of diverse nanoscale perovskite material morphologies, including two-dimensional sheet structures, one-dimensional wire geometries, rod-shaped configurations, zero-dimensional quantum dots, and crystalline nanoparticulate phases. The investigation established that such nanomaterial integrations substantially enhance the electrical–optical characteristics and comprehensive functional performance of perovskite photovoltaic systems.

The study revealed that the incorporation of nanoscale materials addresses several fundamental challenges in perovskite solar cells, including charge transport limitations, light absorption efficiency, and interface quality. The research demonstrated that different nanoscale morphologies offer unique advantages: nanosheets provide enhanced charge transport pathways, nanowires offer improved light scattering and absorption, and quantum dots enable tunable optical properties and enhanced stability.

The advantageous electrical–optical characteristics and simplified manufacturing methodologies of perovskite photovoltaic systems integrated with nanomaterial enhancements establish these configurations as viable candidates for forthcoming photovoltaic technology platforms. The research emphasizes that the strategic selection and integration of appropriate nanomaterials can lead to synergistic effects that enhance multiple performance parameters simultaneously [79].

##### Comprehensive Progress in Efficiency and Stability

Recent comprehensive reviews have highlighted the remarkable progress achieved in perovskite solar cell technology across multiple dimensions. Wu et al. conducted a comprehensive review of the key advancements in perovskite solar cells reported between 2020 and 2021, emphasizing improvements in power conversion efficiency, enhancements in long-term device stability, and progress in the design of perovskite-based tandem architectures.

The review highlights that recent years have witnessed a consistent improvement in both the efficiency and stability of perovskite solar cells, with significant research efforts directed toward minimizing lead leakage and creating environmentally sustainable, lead-free alternatives. This combined emphasis on performance optimization and ecological responsibility marks an essential advancement toward the practical commercialization of perovskite solar technologies.

The research highlights significant progress in perovskite-based tandem devices, which have emerged as one of the most promising pathways for achieving efficiencies beyond the single-junction limit. The development of these devices requires sophisticated interface engineering, optical optimization, and careful material selection to ensure compatibility between different cell components.

Furthermore, the review discusses the ongoing challenges and future prospects of perovskite solar cells, including the development of large-scale manufacturing processes, long-term stability under operational conditions, and the transition from laboratory-scale devices to commercial modules. The research emphasizes that addressing these challenges requires continued innovation in materials science, device engineering, and manufacturing technologies [80].

##### Emerging Applications and Future Directions

In addition to their conventional use in photovoltaic systems, recent studies have examined the adaptability of perovskite materials for next-generation technologies. Korde et al. explored the utilization of perovskite nanostructures in biosensing, showcasing their exceptional multifunctionality beyond solar energy conversion. Their findings indicate that the characteristic structural feature of a central atom coordinated by eight ligands enhances light absorption and charge carrier mobility, thereby making perovskites promising candidates for diverse sensing applications.

Perovskite materials have demonstrated exceptionally high performance in detecting smaller molecules such as O_2_, NO_2_, and CO_2_, leading to the development of several biosensors based on perovskite nanomaterials for detecting various chemical and biological species in both solid and solution states. This research highlights the broader potential of perovskite materials beyond photovoltaics and demonstrates their versatility as functional materials for diverse applications.

The exploration of perovskite materials in biosensor applications also provides insights that can be applied to photovoltaic devices. The understanding of charge transport mechanisms, surface interactions, and stability under various environmental conditions gained from biosensor research can inform the development of more robust and efficient solar cells [81].

##### Challenges and Future Prospects

Despite the remarkable progress made in recent years, several challenges remain to be addressed for the successful commercialization of perovskite solar cells. Afre and Pugliese provided a comprehensive assessment of the current state of the art in perovskite solar cell research, highlighting both achievements and remaining challenges.

The study underscores that, although perovskite solar cells exhibit strong potential as a cost-effective substitute for their traditional silicon-based counterparts, additional research is necessary to enhance their stability under real-world environmental conditions to achieve full commercial viability. Moreover, the mechanical durability of flexible perovskite solar cells has emerged as a crucial research focus, given that flexible configurations open new avenues for applications while simultaneously introducing further stability-related challenges.

Recent investigations have increasingly concentrated on the development of tin-based perovskite solar cells as a promising strategy to address the limitations and environmental concerns linked to lead-containing perovskites. These efforts address both environmental concerns and potential regulatory restrictions on lead-containing materials. The development of lead-free alternatives represents a crucial step toward the widespread adoption of perovskite solar cell technology.

The study further underscores the significance of designing innovative materials for charge transport layers, along with advanced encapsulation methods to shield perovskite solar cells from degradation caused by moisture and oxygen exposure. Such approaches are vital for ensuring the long-term operational stability necessary for successful commercialization [82].

##### Integration of Advanced Characterization Techniques

The progress of perovskite solar cell technology has been greatly facilitated by the adoption of advanced characterization methodologies. The combination of state-of-the-art optical, electrical, and structural analysis techniques has provided researchers with a more profound understanding of the underlying mechanisms that influence device efficiency and long-term stability.

Recent research has demonstrated that the combination of multiple characterization techniques provides a comprehensive understanding of perovskite device operation. This multifaceted approach has been crucial for identifying performance-limiting factors, optimizing device structures, and developing strategies for improving both efficiency and stability.

The application of in situ characterization techniques has been particularly valuable for understanding degradation mechanisms and developing mitigation strategies. These techniques enable real-time monitoring of device performance under various stress conditions, providing insights that are essential for developing robust and stable perovskite solar cells. The continued development and refinement of characterization techniques will be crucial for the future advancement of perovskite solar cell technology as devices become more complex [69].

##### Comprehensive Analysis of High-Efficiency Perovskite Solar Cells

Recent in-depth reviews have offered thorough evaluations of the current developments and future directions of high-efficiency perovskite solar cells. Dastgeer et al. presented a detailed analysis of the key factors influencing the performance of high-efficiency devices, including the intrinsic properties of perovskite materials, device architecture, fabrication methodologies, and the most recent technological advancements. Their study highlights that perovskite solar cells have achieved exceptional progress, attaining a record power conversion efficiency of 25.7%, thereby underscoring their transformative potential within the photovoltaic industry. The study traces the evolution of inverted perovskite solar cells, which made their debut in 2013 with an efficiency of 3.9% and have since achieved significant milestones, including efficiencies exceeding 22.6% by 2021.

The detailed assessment examines key elements crucial to the progression of perovskite solar cell technology, such as stability issues, environmental implications, large-scale production feasibility, and device reproducibility. The study draws attention to persistent challenges associated with material degradation and underscores the need for more robust stability strategies, continued efficiency optimization, and the incorporation of energy storage systems to advance the scalability and manufacturability of perovskite-based technologies.

In addition, the study explores recent developments in tandem and multijunction architectures, as well as the growing interest in flexible and wearable perovskite devices and their incorporation into building-integrated photovoltaic systems. It also offers valuable perspectives on the commercialization prospects of inverted perovskite solar cells, emphasizing that advancements in stability, cost-effectiveness, and efficiency are pivotal for enabling their large-scale adoption and practical deployment [83].

##### Comparative Evaluation of Solar Cell Technologies

An extensive assessment of diverse solar cell technologies has yielded important insights into the competitive position and comparative strengths of perovskite-based devices. Oni et al. carried out a thorough analysis of state-of-the-art photovoltaic technologies, examining emerging materials, loss mechanisms, and strategies for performance optimization across different solar energy platforms.

The analysis covers a wide range of photovoltaic technologies, including silicon and group III–V semiconductors, lead halide perovskites, sustainable chalcogenides, organic solar cells, and dye-sensitized systems, offering a detailed comparison of their respective strengths and challenges. The study points out that, although the efficiency of silicon-based solar cells has reached a plateau near 25%, multijunction solar cells based on III–V compound semiconductors continue to exhibit rising efficiencies; however, their substantial material costs still pose a major limitation to widespread adoption.

The assessment indicates that perovskite solar cells exhibit outstanding efficiency in both single-junction and multijunction configurations, establishing them as strong contenders among existing photovoltaic technologies. Nonetheless, the study identifies several key obstacles that need to be overcome for successful large-scale deployment, including issues related to device degradation, hysteresis behavior, and film uniformity. It further notes that CIGS and CdTe solar cell technologies have shown competitive performance with crystalline silicon cells due to recent improvements; however, environmental concerns and the inherently low open-circuit voltage of CdTe devices continue to pose notable challenges. This comparative evaluation offers a valuable perspective on the technological standing of perovskite solar cells within the broader context of photovoltaic development [84].

##### Roadmap for Perovskite Nanophotonic Applications

The development of perovskite nanophotonics represents an emerging frontier that combines materials synthesis with novel photonic design strategies. Soci et al. presented a comprehensive roadmap for perovskite nanophotonics, outlining the current state and future directions of this rapidly evolving field.

The proposed roadmap incorporates recent progress in synthetic material design, advancements in both bottom-up and top-down nanostructuring techniques, and emerging concepts in nanophotonic engineering aimed at manipulating light–matter interactions at the nanoscale. This interdisciplinary strategy holds considerable promise for influencing present and future technological developments by exploiting the distinctive optical and electronic properties of halide perovskite materials.

The study concludes that the field of perovskite nanophotonics is poised for significant advancement, building upon foundational research on halide perovskite materials that have demonstrated exceptional potential across various optoelectronic and photonic applications. It reflects a unified perspective from leading researchers who have outlined existing challenges and future opportunities while emphasizing the most promising directions for continued exploration in this rapidly evolving domain.

The roadmap functions as an extensive reference for researchers across physics, chemistry, and engineering who are engaged in the study of perovskite nanophotonics, offering valuable direction for future research and technological innovation. The convergence of nanophotonic principles with perovskite materials paves the way for next-generation optical devices, improved light management strategies in solar energy systems, and the development of novel photonic applications.

This roadmap is particularly relevant for the development of advanced perovskite solar cells, as it provides insights into how nanophotonic engineering can be leveraged to improve light absorption, reduce optical losses, and enhance overall device performance. The combination of materials science and photonic design represents a promising pathway for achieving next-generation perovskite photovoltaic devices with superior efficiency and functionality [77].

##### Comprehensive Nanophotonic Design Strategies

Recent comprehensive reviews have highlighted the transformative potential of nanophotonics in advancing perovskite solar cell technology. Furasova et al. provided an extensive analysis of nanophotonic design implementations for enhancing perovskite solar cell efficiency, covering critical methodologies, including nanopatterning, nanotexturing, and nanostructuring approaches [68]. Their work demonstrates how architectural modifications not only optimize optical properties but also significantly influence charge carrier transport and harvesting mechanisms. The review emphasizes the remarkable efficiency progression achieved through nanophotonic integration, with perovskite solar cells advancing from 3.8% efficiency in 2009 to 25.5% for single-junction devices and exceeding 30% for tandem configurations. Notably, the authors highlight the superior integration compatibility of nanophotonic designs with perovskite materials compared to silicon-based systems, which is attributed to the inherent flexibility and defect tolerance of halide perovskites.

The implementation of machine learning techniques for perovskite solar cell design optimization represents another significant advancement covered in this comprehensive review. These computational approaches enable the systematic exploration of design parameters and accelerate the identification of optimal nanophotonic configurations. The work particularly emphasizes the potential for achieving efficiencies approaching the Shockley–Queisser limit of 30–35% through strategic nanophotonic design integration [68].

##### Advanced Photonic Design Principles for Next-Generation Photovoltaics

Garnett et al. presented a broad perspective on photonic design opportunities across various photovoltaic technologies, with significant implications for perovskite solar cell development. Their analysis addresses critical photonic design principles, including nanopatterning methods and metasurfaces for enhanced light incoupling and light trapping in absorber materials [25]. The work identifies key opportunities for reducing carrier recombination through controlled light emission and explores advanced spectral conversion techniques that can be particularly beneficial for perovskite-based systems.

The perspective emphasizes the crucial role of photonic design in next-generation photovoltaic concepts, including tandem and self-adaptive solar cells where perovskites play a central role. The authors address the fundamental challenge of approaching the Shockley–Queisser efficiency limit of 34% through improved photonic design, highlighting how the elimination of losses from incomplete absorption and nonradiative recombination can be achieved through strategic nanophotonic implementation. Their work provides a roadmap for the massive upscaling and integration of photovoltaics, addressing both technical challenges and opportunities in photonic design principles and fabrication methodologies [25].

#### 2.1.3. Critical Discussion and Future Outlook of Nanophotonics for PSC

The rapid evolution of perovskite solar cell (PSC) technology from basic material discovery toward sophisticated nanophotonic engineering has positioned the field at the precipice of commercial viability, yet several critical trade-offs remain unresolved. While laboratory-scale devices have achieved power conversion efficiencies exceeding 25% for single junctions and nearly 30% for tandem configurations, a significant performance divergence persists when transitioning to large-area modules. This “scalability penalty” is exemplified by the marked decrease in efficiency observed when moving from sub-centimeter active areas to decimeter-scale modules, primarily due to the challenges of maintaining film homogeneity and managing increased series resistance over larger substrates. Furthermore, while the integration of periodic nanotextures and metasurfaces has demonstrated substantial optical gains and improved fabrication yields, these nanophotonic enhancements often introduce significant manufacturing complexity. The high-precision lithographic techniques required for such architectures often contrast with the low-cost, solution-processed ethos of perovskite technology, necessitating a move toward high-throughput alternatives like nano-imprinting to balance peak performance with economic feasibility.

Beyond fabrication challenges, the long-term operational stability and environmental impact of PSCs represent the most formidable barriers to widespread adoption. The field faces a persistent tension between the superior optoelectronic performance of lead-based chemistries and the ecological imperative for lead-free alternatives; while tin-based perovskites offer a sustainable pathway, they currently suffer from rapid oxidation and inferior charge transport properties. Innovative strategies such as the deuteration of hole transport materials and the implementation of UV-to-visible photon-converting coatings have bolstered durability, yet their efficacy over a standard twenty-year lifecycle under fluctuating environmental conditions remains empirically unproven. Additionally, the industry has yet to reach a consensus on the optimal tandem architecture, as the integration of two-terminal versus four-terminal configurations involves a fundamental trade-off between simplified external circuitry and the rigorous requirement for precise current matching. Future research must, therefore, prioritize the development of multifunctional nanophotonic designs that simultaneously address light management, moisture encapsulation, and spectral shifting to ensure that PSCs can successfully compete with established silicon-based photovoltaic infrastructures.

### 2.2. Concentrating Solar Power with Light-Trapping Nanostructures

#### 2.2.1. Overview of Concentrating Solar Power Technology

Concentrating solar power (CSP) represents a mature and rapidly evolving solar thermal technology that converts concentrated sunlight into thermal energy, which is subsequently converted to electricity through conventional thermodynamic cycles. Unlike photovoltaic systems that directly convert sunlight to electricity, CSP systems utilize mirrors or lenses to concentrate solar radiation onto receivers, where the concentrated thermal energy heats a working fluid to drive turbines for electricity generation [85,86,87,88].

Nanophotonics has become a groundbreaking strategy for improving the efficiency of CSP systems by enabling precise manipulation of light–matter interactions at the nanoscale. The incorporation of nanophotonic architectures facilitates the creation of spectrally selective absorbers with finely tuned optical characteristics, capable of achieving solar absorptance levels above 95% while keeping infrared emissivity below 4%, even under high-temperature operating conditions [89].

#### 2.2.2. Recent Developments in Concentrating Solar Power

##### Femtosecond Laser Nanostructuring for Enhanced Solar Absorption

Recent advances in ultrashort laser pulse technology have enabled the development of sophisticated nanostructured surfaces for CSP applications. Santagata et al. demonstrated significant improvements in solar absorptance through femtosecond laser texturing of commercial molybdenum surfaces [85]. Their work achieved solar absorptance values approximately four times higher than pristine molybdenum samples at 800 K, with maximum improvements observed for surfaces treated at intermediate laser fluences (1.8 to 14 J/cm^2^).

The enhancement mechanism relies on laser-induced periodic surface structures (LIPSSs) that create subwavelength periodicity patterns on the molybdenum surface. These nanostructures function as light-trapping elements, significantly increasing the effective absorption area and surface roughness. The study revealed that LIPSS formation results from interference between incident laser waves and surface plasmon polaritons, leading to periodic ripples with periodicities comparable to or smaller than the laser wavelength.

Thermal stability testing demonstrated that the nanostructured molybdenum surfaces maintain their enhanced optical properties even after prolonged thermal annealing at operating temperatures typical of thermionic converters. The research established that intermediate laser fluences provide optimal performance by balancing enhanced light trapping with controlled oxide formation, while excessive fluences lead to significant oxide presence that reduces selectivity despite maintaining high absorptance [85].

##### Multi-Scale Light-Trapping Nanostructured Coatings

The development of light-trapping nanostructured coatings represents a breakthrough approach for next-generation CSP receivers operating at elevated temperatures. Wang et al. developed a comprehensive multi-scale modeling framework combining Monte Carlo Ray Tracing (MCRT), the Finite Difference Time Domain (FDTD), and the Finite Volume Method (FVM) to evaluate receiver performance across nine orders of magnitude, from heliostat fields (~10 m) to nanostructured coatings (~100 nm).

Their investigation of three distinct nanostructure geometries—pyramid, moth-eye, and cone structures—revealed that cone nanostructures achieve superior optical–thermal performance with receiver efficiencies exceeding 88%. This represents a 6–10 percentage point improvement over commercial Pyromark 2500 coatings. The enhanced performance stems from optimized light-trapping mechanisms that increase solar absorption while maintaining low thermal emittance.

The multi-scale modeling approach successfully bridged the gap between nanoscale optical phenomena and system-level performance, enabling accurate prediction of how nanostructured coatings influence overall CSP plant efficiency. The study demonstrated that metallic nickel-based nanostructured arrays provide intrinsically low emissivity while achieving high solar absorption through carefully engineered surface texturing [86].

##### High-Performance Multilayer Selective Solar Absorbers

Advanced multilayer selective solar absorber configurations have emerged as promising solutions for high-temperature CSP applications. Farchado et al. developed a novel six-layer selective absorber (SiO_2_/PtAl_2_O_3_/Pt/PtAl_2_O_3_/CuCoMnOx/SiO_2_) designed for operation at temperatures up to 550 °C in air atmosphere, addressing the critical challenge of oxidation-induced degradation in next-generation CSP systems [87].

The optimized multilayer configuration demonstrated outstanding optical characteristics, attaining a solar absorptance of 0.957 and a thermal emittance of 0.10 at 500 °C when fabricated on stainless steel 316L substrates through an economical dip-coating process. The design philosophy incorporates platinum-based intermediate layers that provide oxidation resistance while maintaining high optical performance, with copper–cobalt–manganese oxide (CuCoMnOx) as the primary absorbing layer.

Durability testing demonstrated outstanding thermal stability, with the multilayer stack withstanding 3072 h at 500 °C in open-air atmosphere without performance degradation (PC < 0.01). The material’s resistance to condensation conditions and vacuum loss makes it particularly suitable for parabolic trough receivers, where maintaining performance during operational transients is critical. XRD analysis confirmed high crystallinity of constituent layers, while XPS depth profiling verified the integrity of the six-layer sequence [87].

##### Plasmonic Meta-Structure Solar Absorbers

Significant advancements have been made in the design of plasmonic nanostructures for broadband solar absorption by leveraging the synergistic interaction of multiple absorption mechanisms. Su et al. proposed a high-efficiency metastructure solar absorber (MSSA) composed of tungsten truncated cone arrays integrated with metal–insulator–metal (MIM) resonator layers, achieving enhanced optical performance across a broad spectral range [89].

The optimized structure demonstrated outstanding performance, achieving a total solar absorption efficiency above 97.1% and a thermal emissivity below 8.5% under one-sun illumination, corresponding to a photothermal conversion efficiency of 91.6% at 100 °C. Its broadband absorption extended across the ultraviolet, visible, and near-infrared spectrum (280–1700 nm), maintaining an absorptance greater than 97.8% throughout this wide spectral range.

**Figure 4:**
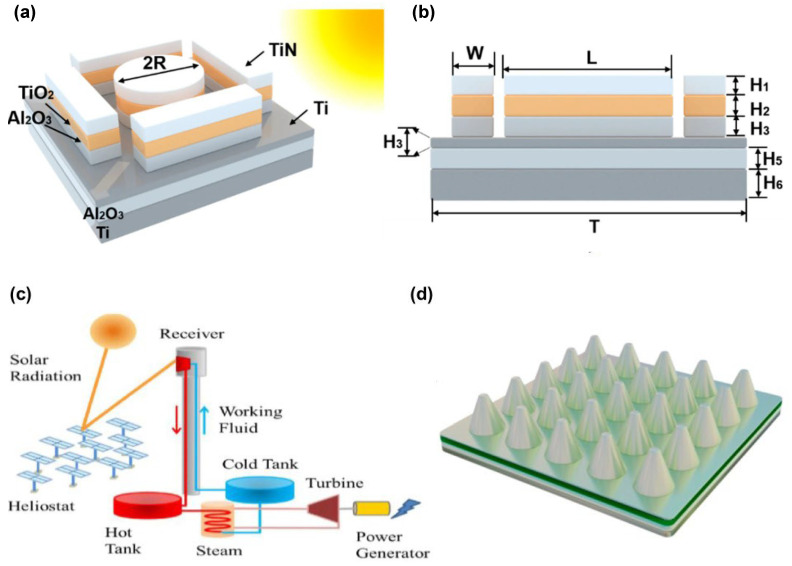
Selected recent developments in nanophotonic strategies aimed at improving the efficiency of CSP collectors. (**a**) Solar receiver incorporating a metamaterial-based absorber. (**b**) Two-dimensional periodic structural design of the receiver [27]. (**c**) Schematic illustration of a receiver system generating electrical power [88]. (**d**) 3D schematic of the metastructure solar absorber (MSSA) [89].

The superior absorption characteristics arise from the combined influence of several mechanisms, including magnetic polaritons (MPs) generated on the nanostructured tungsten surface, cavity plasmon resonances formed between the truncated cone arrays that enhance light trapping, magnetic field resonances within the MIM optical resonator, and the intrinsic optical losses of the tungsten itself. The impedance matching with free space ensures efficient light coupling, while the tungsten material provides exceptional thermal stability with a melting point around 3400 °C (Figure 4) [89].

##### Four-Pointed Star Metamaterial Absorbers for High-Temperature Applications

Advanced metamaterial absorber architectures have shown remarkable promise for efficient solar energy harvesting at elevated temperatures. Qiu et al. introduced a selective metamaterial absorber incorporating four-pointed star prism geometries, specifically engineered to maintain stable performance under concentrated solar irradiation at temperatures reaching up to 1673 K.

The optimized absorber exhibited outstanding spectral selectivity, achieving a total solar absorptance of 0.958 and total emittance values between 0.2355 and 0.4062, corresponding to solar-to-thermal conversion efficiencies ranging from 92.31% to 77.78% under 1000-sun concentration at operating temperatures between 1273 and 1673 K. The strong solar-band absorptance arises from effective impedance matching and the coupling of multiple plasmonic modes, whereas the suppressed mid-infrared emittance is attributed to intentional impedance mismatching that minimizes radiative losses.

Parametric studies revealed that specific geometric parameters, including the height of the SiO_2_ layer above the star structure and the short diagonal length of the rhomb, have minimal influence on spectral absorptance within certain ranges, providing design flexibility for manufacturing tolerances. The absorber demonstrates excellent insensitivity to polarization angle and incident angle variations, making it suitable for practical CSP applications where solar tracking precision may vary.

The research established that refractory metals like tungsten provide optimal characteristics for high-temperature metamaterial absorbers due to their high absorptance in the near-infrared wavelength range resulting from interband transitions, combined with exceptional thermal stability. These properties make four-pointed star metamaterial absorbers promising candidates for next-generation CSP systems operating at ultra-high temperatures [90].

##### One-Dimensional Multilayer Nanostructures for High-Temperature Applications

Yuan et al. developed a high-temperature solar selective absorber based on simple one-dimensional multilayer nanostructures, addressing the critical challenge of balancing thermal performance with structural complexity in concentrated solar power systems [91]. The research focused on creating a cost-effective solution that maintains excellent performance while reducing fabrication complexity compared to existing three-dimensional metamaterial structures.

The proposed absorber consists of alternating layers of tungsten (W) and silicon dioxide (SiO_2_) in a one-dimensional multilayer configuration. The optimal design features ten layers with specific geometric parameters: h_1_ = 90 nm, h_2_ = 5 nm, h_3_ = 80 nm, h_4_ = 7 nm, h_5_ = 80 nm, h_6_ = 5 nm, h_7_ = 60 nm, h_8_ = 5 nm, h_9_ = 60 nm, and h_10_ = 200 nm. The material selection of tungsten (with a melting point of 3422 °C) and silicon dioxide (with a melting point of 1723 °C) ensures thermal stability under extreme operating conditions [91].

The absorber exhibits outstanding performance characteristics, achieving a total solar absorptance of 0.9504 under AM1.5 solar illumination. When operated at 1000-sun concentration at 1273 K, it attains an impressive solar-to-thermal conversion efficiency of 91.2%, accompanied by a low total emittance of 0.1504. Moreover, the structure maintains excellent stability across varying polarization states and wide incident angles, rendering it highly suitable for practical CSP systems where solar tracking accuracy may fluctuate.

Comprehensive performance evaluation across concentration coefficients ranging from 300 to 1300 suns and temperatures from 500 to 1500 K confirmed the absorber’s robust operation under diverse conditions. Analysis of the spectral selectivity mechanism indicated that the outstanding absorption performance stems from effective impedance matching, which arises from the coupled interaction between surface plasmon polaritons and magnetic polaritons [91]. This fundamental understanding provides insights for further optimization of one-dimensional nanostructured absorbers.

The fabrication advantages of this design are significant, as the one-dimensional multilayer structure can be manufactured using cost-effective magnetron sputtering coating techniques, in contrast to the expensive photolithography and micro–nano-coating processes required for complex three-dimensional metamaterial structures. This approach substantially reduces manufacturing costs while maintaining high performance, making it more viable for large-scale CSP deployment.

#### 2.2.3. Critical Discussion and Future Outlook of Nanophotonics for CSP

The advancement of nanophotonic strategies in CSP marks a critical transition from conventional blackbody-like absorbers toward spectrally selective surfaces designed to maximize solar harvesting while suppressing radiative thermal losses. A comparative evaluation of current methodologies reveals a fundamental trade-off between peak optical performance and manufacturing scalability. While complex three-dimensional metamaterials, such as tungsten-based truncated cones and four-pointed star prisms, achieve exceptional solar absorptance (exceeding 97%) and high photothermal conversion efficiencies at ultra-high temperatures, their reliance on high-precision lithography poses a significant barrier to large-scale deployment. In contrast, one-dimensional multilayer stacks and femtosecond laser-induced periodic surface structures (LIPSSs) offer a more pragmatic pathway for industrial scaling due to their compatibility with cost-effective techniques like magnetron sputtering and high-throughput laser processing. However, these simpler architectures often face challenges in maintaining perfect impedance matching across the entire solar spectrum, resulting in a slightly lower efficiency than their 3D counterparts, which underscores a persistent need for designs that balance architectural simplicity with broadband selectivity.

Beyond the initial optical efficiency, the long-term operational viability of nanostructured CSP absorbers is dictated by the rigorous demands of thermal stability and oxidation resistance at temperatures reaching up to 1673 K. The integration of refractory metals like tungsten and molybdenum provides an essential foundation for high-temperature durability; however, the vulnerability of these materials to atmospheric oxidation requires the development of sophisticated protective encapsulants, such as the six-layer SiO_2_/PtAl_2_O_3_ configurations discussed. While platinum-based intermediate layers significantly enhance oxidation resistance, they introduce substantial material costs that may limit the overall economic competitiveness of the technology. Furthermore, a critical unresolved challenge remains the mechanical integrity of these nanostructures under the severe thermal cycling and transient conditions typical of real-world solar fields. Future research must move toward bridging the gap between multi-scale computational modeling and long-duration field testing to ensure that the impressive laboratory-scale gains in spectral selectivity can be maintained over a twenty-year service life in oxidizing environments.

### 2.3. Nanophotonic-Based Solar Thermophotovoltaics

#### 2.3.1. Overview of Solar Thermophotovoltaic Technology

Solar thermophotovoltaics (STPV) represents an innovative approach to solar energy conversion that has garnered significant attention due to its potential to exceed the Shockley–Queisser limit of conventional single-junction photovoltaic cells [92]. Unlike direct photovoltaic conversion, STPV systems employ a two-step energy conversion process: first converting concentrated solar radiation into thermal energy and subsequently converting thermal radiation from a heated emitter into electricity using photovoltaic cells [93]. This intermediate thermal step enables spectral control and optimization opportunities that can theoretically achieve conversion efficiencies exceeding 50% [94,95,96,97,98].

The role of nanophotonics in STPV systems has become increasingly critical as researchers seek to approach theoretical efficiency limits. Nanophotonic structures enable unprecedented control over thermal emission characteristics through engineered optical resonances, surface plasmon effects, and photonic bandgap phenomena. These capabilities allow the design of selective emitters with precisely tailored spectral properties, enhanced absorption and emission characteristics, and improved thermal stability. Photonic crystals, metamaterials, and metasurfaces have emerged as particularly promising approaches for achieving the spectral selectivity required for high-efficiency STPV operation [99].

#### 2.3.2. Recent Developments in Solar Thermophotovoltaics

The integration of nanophotonics with solar thermophotovoltaic systems has witnessed remarkable progress in recent years, with significant advances in selective emitter design, spectral control, and system efficiency. The following subsections detail the latest developments in STPV nanophotonics based on cutting-edge research from 2020–2025.

##### Nanostructured Multilayer Selective Emitters

Bhatt et al. demonstrated a high-efficiency STPV system employing a nanostructure-based selective emitter with multilayer metal–dielectric coatings [96]. The selective emitter consisted of Si_3_N_4_/W multilayer nanostructures deposited on a tungsten substrate, designed to achieve optimal spectral control for GaSb-based TPV cells. The experimental setup produced an electrical power density of 1.71 W/cm^2^ at an operating temperature of 1676 K, corresponding to an overall power conversion efficiency of 8.4% when normalized to the emitter surface area. This value represented the highest STPV efficiency reported at the time, underscoring the effectiveness of nanostructured selective emitters in significantly improving system performance. The multilayer design enabled precise control over the spectral emission characteristics, maximizing the overlap between the emitter spectrum and the TPV cell’s spectral response while minimizing sub-bandgap losses.

##### Comprehensive Nanostructure Design Strategies

Gupta and Bhatt presented a comprehensive review of micro- and nanostructured selective absorber and emitter surfaces designed to enhance the efficiency of solar thermophotovoltaic (STPV) systems [95]. Their work examined various nanostructure types, including random textures, nanocones, nanoholes, and multilayer metal–dielectric stacks, demonstrating how these structures create interference effects for photons with wavelengths comparable to the feature sizes. Experimental validation using a Si_3_N_4_-W-Si_3_N_4_ selective emitter achieved an overall power conversion efficiency of 8.6% at 1670 K. The study emphasized that spectral selectivity through nanostructures enables STPV systems to surpass the Shockley–Queisser limit by tailoring the incident spectrum to match the TPV cell’s bandgap, with theoretical efficiencies approaching 45% for optimized systems.

##### Tamm Plasmon-Enabled Narrowband Thermal Emitters

**Figure 5:**
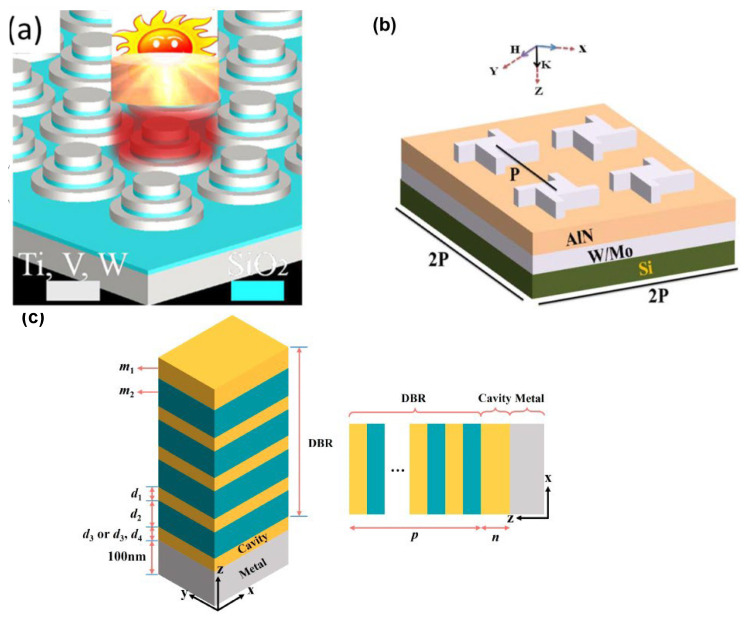
Representative of recent advancements in nanophotonic approaches for improving solar thermophotovoltaic (STPV) performance. (**a**) Schematic illustration of a W/SiO_2_/W structure incorporating periodic truncated or full nanocones. Redrawn from Ref. [97]. (**b**) Three-dimensional nano-grating architecture based on a metal–dielectric–metal (MDM) configuration [98]. (**c**) Optical characteristics of an OTS-based emitter, including a multilayer structural diagram with detailed geometric parameters [99].

Lin et al. designed a spectrally selective thermal emitter utilizing optical Tamm states (OTSs) within a one-dimensional photonic crystal structure. The emitter was composed of alternating layers of HfO_2_ and SiO_2_ deposited on a molybdenum substrate, achieving a peak simulated thermal emissivity of 0.97 with an ultra-narrow bandwidth of 48 nm centered at a wavelength of 1.9 μm. When integrated into an STPV system, the proposed emitter delivered an overall efficiency of 33.7% at a solar concentration of 2500 and an emitter-to-cell area ratio of 20. The optical Tamm state mechanism enabled precise spectral control by confining electromagnetic fields at the interface between the metallic substrate and the photonic crystal structure, demonstrating the potential of quantum optical phenomena for advanced STPV applications (Figure 5).

##### Nanolayered Wavelength-Selective Emitters

Wang et al. presented nanolayered narrowband thermal emitters utilizing Tamm plasmon polaritons (TPPs) in a-SiN_x_ and a-SiN_γ_O_γ_ alternatively stacked nanolayers [100]. The emitters were constructed on polished silicon substrates coated with a metallic molybdenum layer, displaying narrowband absorption with an absorptance exceeding 90% at the target emission wavelength. Simulations indicated that the corresponding STPV system could achieve an efficiency of 28.9% at a solar concentration of 1000. A notable advantage of the molybdenum-based configuration was its minimal absorption—reduced to approximately 1.4%—within the 2–7 μm spectral range, along with its excellent thermal stability under high-temperature conditions in an air environment. The tunability of absorption spectra through simple thickness variation of the multilayers provided design flexibility for different TPV cell bandgaps.

##### Dual-Coherence Enhanced Absorption Systems

Zhang et al. proposed an innovative spectrally selective, high-temperature-resistant thermal emitter based on the concept of dual-coherence enhanced absorption (DCEA) [101]. The structure consisted of a multilayer Si/Mo/AlN lamellar film deposited on a molybdenum substrate via sequential physical vapor deposition. The emitter achieved a peak emissivity of approximately 97% at 1.4 μm while effectively suppressing emission to about 10% within the 3.4–10 μm wavelength range, maintaining stable performance up to 973 K. When integrated into STPV systems, the DCEA-based emitter led to a 20% improvement in overall system efficiency compared with single-mode coherent perfect absorbers, highlighting the benefits of multi-path optical interference for refined spectral control.

##### Metamaterial Selective Emitters for High-Bandgap Cells

Tian et al. developed a metamaterial-based selective emitter composed of tantalum and Al_2_O_3_, specifically engineered to optimize performance for high-bandgap silicon photovoltaic cells [102]. In a concentrated STPV configuration integrating a cavity-structured absorber and the proposed metamaterial emitter, the system achieved an efficiency of 37.18%. Notably, the proportion of the usable spectrum for silicon cells increased substantially—from 16.52% to 72.69%—as a result of spectral reshaping enabled by the selective emitter. The findings revealed that STPV systems employing silicon cells outperformed those using lower-bandgap materials such as GaSb by approximately 12–13 percentage points, underscoring the promise of high-bandgap photovoltaic technologies when combined with advanced nanophotonic spectral management.

##### Non-Hermitian Selective Thermal Emitters

Prasad and Naik developed a groundbreaking approach using non-Hermitian optics for selective thermal emission [103]. Their hybrid metal–dielectric non-Hermitian selective emitter (NHE) exhibited outstanding spectral efficiency exceeding 60% and achieved a peak thermophotovoltaic (TPV) conversion efficiency of 12% at an operating temperature of 1273 K. The non-Hermitian approach represents a novel quantum-inspired methodology for controlling thermal emission, offering new possibilities for enhancing spectral selectivity beyond conventional approaches. The work addressed the fundamental limitations of traditional selective emitters by leveraging non-Hermitian optical phenomena to achieve superior performance characteristics.

##### Nanoscale Grating Metamaterial Emitters for High-Temperature Applications

Feyisa et al. developed nanoscale grating metamaterial emitters based on tungsten/molybdenum films with aluminum nitride spacers for thermophotovoltaic applications [98]. The three-dimensional metal–dielectric–metal (MDM) grating configurations demonstrated outstanding emissive performance, with the W–AlN–W structure achieving an average emittance of 94% over the 0.3–2.2 μm wavelength range and its Mo–AlN–Mo counterpart reaching 93% across 0.3–2.0 μm at normal incidence. The optimized emitter designs attained high spectral efficiencies of 87% and 87.5% at 1600 K for InGaAs photovoltaic cells with bandgaps of 0.55 eV and 0.62 eV, respectively. These metamaterial emitters also exhibited polarization-independent behavior and maintained strong emissivity across a broad range of incident angles (0–75°). Their superior performance was primarily attributed to the combined influence of surface plasmon polaritons, magnetic polaritons, and the inherent absorption characteristics of the metallic components. The design offers advantages of high thermal stability, easy fabrication, cost-effectiveness, and the unique capability of using one structure for two different bandgaps.

##### Near-Field Thermophotovoltaic Devices

Song et al. explored the performance potential of multijunction photovoltaic cells in near-field thermophotovoltaic (NF-TPV) systems, with particular attention to photon tunneling phenomena and scalability aspects. Their comprehensive analysis evaluated the additional losses associated with high photocurrent densities and introduced approximate analytical models to quantify these effects. The study offered valuable insights into the scalable design of NF-TPV systems, highlighting the capacity of multijunction architectures to boost power output density through enhanced near-field radiative heat transfer. The proposed analytical framework allows for accurate performance prediction in devices incorporating ten or more subcells, enabling the systematic optimization of parameters such as vacuum gap spacing and emitter temperature.

##### Metasurface-Controlled Thermal Emission

Chu et al. provided a comprehensive review of controlling thermal emission with metasurfaces and their applications in thermophotovoltaic systems [104]. The review highlighted recent advances in tuning thermal emission across multiple degrees of freedom, including wavelength, polarization, radiation angle, and coherence, using two-dimensional subwavelength artificial nanostructures. Metasurfaces offer unprecedented flexibility in shaping thermal emission characteristics, enabling compact and integrated optical devices for STPV applications. The work emphasized the transition from broadband, unpolarized, and incoherent conventional thermal emission to precisely controlled emission through metasurface engineering.

##### Chromium Metasurface Broadband Absorbers

Rana et al. designed a broadband metasurface solar absorber made from refractory chromium for use in intermediate structures of STPV systems [105]. The metasurface demonstrated outstanding broadband absorptance, maintaining an average above 90% throughout the 300–1200 nm spectral range. The corresponding STPV configuration achieved a photovoltaic cell efficiency of 43.2%, with performance remaining above 42% across a wide color temperature range of 1597–2573 K. The use of chromium endowed the absorber with inherent self-passivating behavior, offering strong resistance to oxidation and corrosion, as well as excellent thermal stability and cost-effectiveness. This advancement was driven by enhanced efficiency resulting from the synergistic integration of spectral selectivity and broadband optical response in both the absorber and emitter components.

##### Nanocone-Based Photonic Crystal Absorbers

Mirnaziry et al. examined the optical and thermal performance of two-dimensional photonic crystal absorbers consisting of tungsten nanocone arrays, specifically designed for solar thermophotovoltaic (STPV) applications [104]. The study examined both complete and truncated nanocone shapes, analyzing their thermo-optical performance through comprehensive modeling. The nanocone-based design leveraged photonic crystal effects to enhance solar absorption while maintaining thermal stability at high operating temperatures. The research provided insights into optimizing nanocone geometries for maximum absorption efficiency and thermal management in practical STPV systems.

##### Optically Transparent Metasurface-Based STPV Systems

Shafique et al. proposed an innovative optically transparent metasurface (OTM)-based STPV system that successfully combines high solar energy conversion efficiency with visible transparency [106]. The design utilizes indium tin oxide (ITO) as a transparent conductive metal and zinc sulfide (ZnS) as the substrate, forming a four-layer configuration featuring cross-shaped resonators. This OTM architecture exhibits outstanding broadband absorption performance, achieving up to 99% absorption in the ultraviolet range (250–400 nm) and over 90% absorptivity in the far-infrared region (800–2000 nm) while maintaining excellent visible-light transmittance. Such a balance enables effective solar energy harvesting without compromising transparency, making it particularly suitable for building-integrated photovoltaic (BIPV) applications. The metasurface also demonstrates exceptional angular stability, sustaining absorption rates above 90% for incident angles up to 70° under both TE and TM polarizations. The transparent nature of the structure offers significant potential for integration into architectural glass, vehicle surfaces, and portable electronics, with the capability to offset over 40% of a building’s energy consumption when implemented on window exteriors. The study further establishes that transparent conducting oxides such as ITO can act as efficient plasmonic materials for STPV applications, providing a scalable, low-cost pathway toward aesthetically compatible renewable energy solutions.

#### 2.3.3. Critical Discussion and Future Outlook of Nanophotonics in STPV

The field of STPV has undergone a fundamental transition from basic thermal-to-electric conversion toward high-precision spectral management enabled by nanophotonic engineering. A comparative evaluation of current strategies reveals that, while multilayer metal–dielectric stacks and one-dimensional photonic crystals (employing Tamm plasmon polaritons) offer exceptional narrowband emission, they often require extreme solar concentration factors—frequently exceeding 1000 to 2500 suns—to reach their theoretical efficiency peaks. This high concentration requirement introduces a significant trade-off between peak system efficiency (reaching up to 37.18% in high-bandgap silicon systems) and the thermal management complexity required to prevent component degradation. Furthermore, while quantum-inspired approaches like non-Hermitian optics and dual-coherence enhanced absorption provide unprecedented control over thermal emission bandwidth, their implementation often involves intricate physical vapor deposition sequences that may challenge large-scale manufacturability. The shift toward high-bandgap photovoltaic cells, such as silicon, underscores a strategic move to utilize materials with higher open-circuit voltage potential, yet this necessitates even more rigorous spectral tailoring to minimize the massive thermalization losses inherent in high-energy photon harvesting.

Despite these advances, the path to commercial STPV deployment is obstructed by persistent challenges in material durability and system-level integration. Although refractory metals like tungsten and molybdenum, along with self-passivating materials like chromium, have demonstrated impressive stability at temperatures exceeding 1000 K, the long-term structural integrity of nanoscale features under continuous high-temperature operation remains a primary concern. Surface diffusion and grain growth at elevated temperatures can alter the precise geometries of nanocones and gratings, leading to spectral “drift” and a subsequent decline in efficiency over time. Furthermore, the emerging frontier of near-field TPV offers a theoretical pathway to surpass the Blackbody limit through photon tunneling, yet maintaining subwavelength vacuum gaps over large areas represents a formidable mechanical engineering hurdle that has yet to be solved for industrial scales. Conversely, the development of optically transparent metasurfaces for building-integrated applications introduces a new trade-off between visible-light transmittance and ultraviolet/infrared harvesting efficiency. Future research must prioritize the development of “thermally robust” nanophotonics—structures that maintain their prescribed optical response through thousands of thermal cycles—while simplifying fabrication protocols to ensure that STPV systems can achieve a competitive levelized cost of energy.

## 3. Nanophotonics for Biosensing Applications

Nanophotonics has emerged as a transformative field in biosensing, offering unprecedented sensitivity, real-time operation, miniaturization, and multiplexing capabilities addressing critical gaps in diagnostics, environmental monitoring, and food safety. By leveraging light–matter interactions at the nanoscale, these biosensors enable rapid, label-free detection of biomolecules, pathogens, and environmental contaminants. This review synthesizes insights from 40+ recent studies (2018–2024) to examine the key material innovations (plasmonic metals, 2D materials, and hybrids) and their performance trade-offs; advantages over conventional techniques (e.g., ELISA and PCR); applications across clinical, environmental, and industrial sectors; challenges (fabrication and scalability); and future directions (AI integration and wearables).

### 3.1. Key Nanophotonic Materials for Biosensors

**Figure 6:**
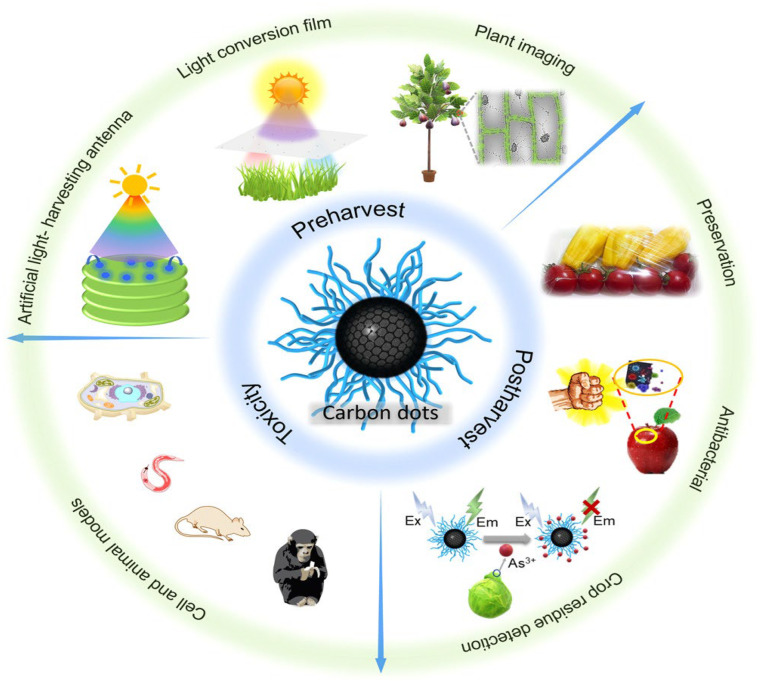
Carbon dot nanosensors: A visual exploration of revolutionary applications. Reprinted and permission from Ref. [118].

**Figure 7:**
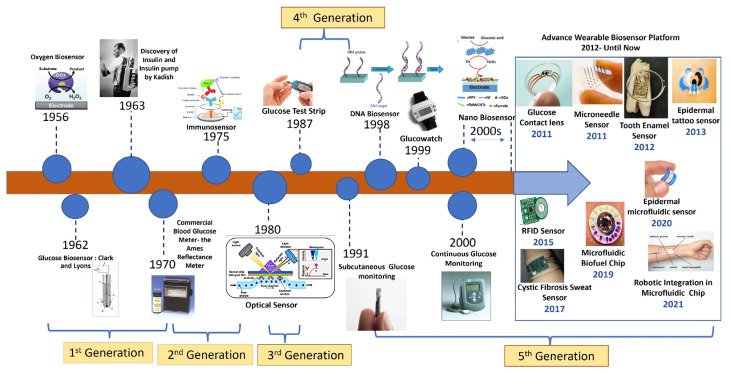
The timeline highlights key technological milestones and different generations in the convergence of nanosensors with multi-omics platforms, including electrical, optical, and chemical nanosensors. Adapted from Ref. [125] with permission from MDPI.

The performance of nanophotonic biosensors is heavily influenced by the choice of materials, which determine sensitivity, stability, and compatibility with biological samples. These materials are categorized as follows: (1) Plasmonic metals, such as gold (Au) and silver (Ag), are widely used due to their strong localized surface plasmon resonance (LSPR), which enhances electromagnetic fields for ultrasensitive detection. For example, Au-Ag bimetallic nanoparticles (NPs) amplify surface-enhanced Raman scattering (SERS) signals by 14 fold, enabling single-molecule detection of viruses [105,106], while Ag nanocubes show 10× higher SERS signals than spheres but require SiO_2_ coatings to prevent oxidation [107]. Aluminum (Al) extends plasmonics into the ultraviolet range, facilitating applications like H. pylori detection in clinical samples [108]. (2) Dielectric materials, including silicon nitride (Si_3_N_4_) and titanium dioxide (TiO_2_), are prized for their low optical losses and compatibility with complementary metal–oxide–semiconductor (CMOS) technology. Si_3_N_4_ waveguides, for instance, achieve refractive index resolutions of 10^−8^–10^−7^ RIU, making them ideal for detecting SARS-CoV-2 at 200 copies/μL without amplification [109]. TiO_2_ nanosheets, on the other hand, combine sensing with photocatalytic pathogen inactivation, adding a therapeutic dimension to biosensing [110]. (3) Two-dimensional (2D) materials, such as molybdenum disulfide (MoS_2_) and graphene, offer unique electronic and optical properties. MoS_2_-based field-effect transistors (FETs) detect miRNA155 at concentrations as low as 0.03 fM, while graphene oxide aptasensors identify aflatoxin B_1_ in food at 0.25 ng/mL, surpassing traditional ELISA methods [111,112]. (4) Carbon-based nanomaterials, including carbon quantum dots (CQDs) and carbon nanotubes (CNTs), provide biocompatibility and versatility (Figure 6). Nitrogen-doped CQDs synthesized from radish leaves detect the anticancer drug nintedanib at 0.14 μg/mL via fluorescence quenching, showcasing their potential for environmental monitoring [113]. CNTs, with their high aspect ratio and conductivity, are employed in electrochemical biosensors for real-time glucose monitoring in urine [114]. (5) Hybrid materials, such as metal–organic frameworks (MOFs) and core–shell nanoparticles, combine the strengths of multiple components. ZIF8 MOFs stabilize plasmonic gold nanorods (Au-NRs), enabling glyphosate detection in water, while silica-coated silver nanoparticles (Ag@SiO_2_) reduce quenching in SERS tags, improving signal reproducibility [105,115]. (6) Emerging materials, like rare-earth-doped NPs and chiral metasurfaces, address niche applications. Rare-earth elements (e.g., europium and dysprosium) enhance electrochemical biosensors for neurotransmitters, while chiral metasurfaces enable enantiomer-specific detection of biomolecules, critical for pharmaceutical applications (Figure 7) [116,117].

### 3.2. Advantages of Nanophotonics in Biosensing

Nanophotonic biosensors offer several advantages over conventional techniques, including the following: (1) Ultra-high sensitivity is a hallmark of nanophotonics, with LSPR biosensors detecting cardiac troponin I (cTnI) at 0.01 ng/mL and SERS achieving single-molecule resolution [107,119]. Photonic crystal sensors further push the limits, resolving interleukin6 (IL6) at 10 pg/mL in wound exudate, enabling early diagnosis of chronic infections [120]. (2) Rapid and label-free operation eliminates the need for time-consuming sample preparation and fluorescent labeling. For example, silicon nitride (Si_3_N_4_) interferometers quantify whole SARS-CoV-2 viruses in under 20 min, rivaling PCR accuracy without amplification [109]. Similarly, plasmonic gold nanoparticles (AuNPs) provide real-time monitoring of thrombin/DNA interactions, crucial for studying coagulation disorders [111]. (3) Multiplexing allows the simultaneous detection of multiple analytes, a critical feature for complex diagnostics. Quantum dot barcodes distinguish nine respiratory viruses in a single assay, while TriPlex^TM^ photonic waveguides monitor pollutants like bisphenol A (BPA) and atrazine in water with limits of detection (LODs) as low as 0.06 µg/L [121,122].

Furthermore, portability and integration with point-of-care (POC) devices are facilitated by miniaturization. Smartphone-coupled AuNP assays detect pesticides in food with visual readouts, and 3D-printed graphene electrodes enable decentralized glucose monitoring [114,123], while artificial intelligence (AI) integration is an emerging trend, enhancing data analysis and sensor performance. Deep learning models, such as convolutional neural networks (CNNs), reduce SERS spectral artifacts by 82%, enabling accurate classification of lung cancer biomarkers [111,124].

### 3.3. Applications of Nanophotonic Biosensors

**Figure 8:**
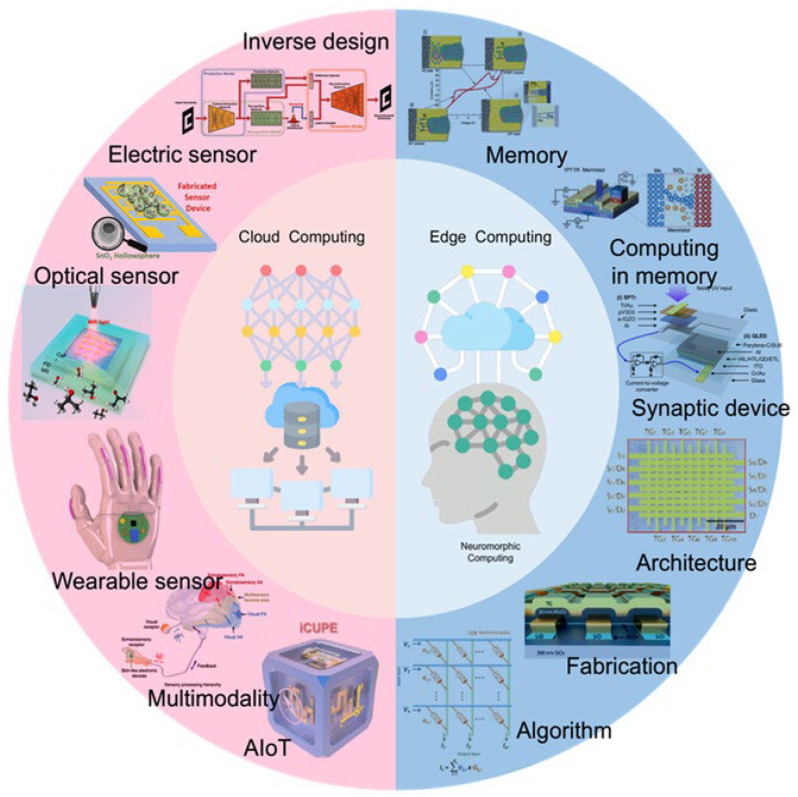
Scheme of machine learning-assisted nanosensor arrays and their applications in wearable electronics. Reprinted with permission from Ref. [127].

Nanophotonic biosensors have transcended laboratory research to become pivotal tools in real-world scenarios, revolutionizing detection capabilities across multiple disciplines. Their unparalleled sensitivity, multiplexing capacity, and miniaturized form factors enable transformative applications in precision medicine, global health surveillance, environmental protection, and food security (Figure 8).

#### 3.3.1. Biomolecule Detection

These biosensors excel in identifying proteins, nucleic acids, and small molecules, which is critical for the following: (1) Early disease diagnosis: Detection of low-abundance biomarkers (e.g., cardiac troponin I at 0.01 ng/mL [119]) enables timely intervention for conditions like myocardial infarction. (2) Epigenetic research: FRET-based quantum dots (QDs) map DNA methylation patterns (e.g., hypermethylation of tumor suppressor genes [126]) with 10× higher resolution than microarrays, advancing personalized cancer therapies. (3) Point-of-care metabolic monitoring: Catalytic MOFs (e.g., Ce-MOFs [115]) provide continuous glucose tracking without finger-prick blood sampling, addressing a key need for 463 million diabetics worldwide. Traditional techniques (e.g., Western blotting) require 24+ hours and lose sensitivity for rare biomarkers—a gap that nanophotonics closes.

#### 3.3.2. Medical Diagnostics

Nanophotonic biosensors are transforming modern medicine by enabling earlier, more accurate disease detection than conventional methods. In infectious disease detection, the PANTOMIM biosensor identifies urinary tract infections (UTIs) at 1.23 CFU/mL, and SERS molecular beacons diagnose methicillin-resistant *Staphylococcus aureus* (MRSA) within 45 min [105,128]. For cancer, Au-MoS_2_ nanosheets detect carcinoembryonic antigen (CEA) at 1.6 fg/mL [112]—equivalent to finding 1 cancerous cell among 10^9^ healthy ones—potentially reducing late-stage cancer mortality by 50%, as well as enabling early intervention. Chronic disease management benefits from guided-mode resonance (GMR) sensors that track IL6 in chronic wounds, predicting healing trajectories [120].

#### 3.3.3. Environmental Monitoring

The unprecedented sensitivity of nanophotonic biosensors is addressing critical gaps in environmental surveillance. In mitigating water pollution caused by heavy metal toxicity, water quality assessment employs photonic crystal fibers to detect cadmium (Cd^2+^) at 3.8 × 10^−11^ M, while hyperspectral imaging identifies airborne pathogens in real time [110,112].

#### 3.3.4. Food Safety

Nanophotonic biosensors are becoming essential tools for ensuring food safety from production to consumption. They can be used for pathogen detection, toxin monitoring, and assessment of supply chain integrity. AuNP-based lateral flow assays (LFAs) screen for E. coli O157:H7 in meat, and SERS nanotags quantify ochratoxin A in grains, ensuring compliance with safety standards [129,130].

### 3.4. Challenges and Future Directions

Despite their promise, nanophotonic biosensors face several challenges such as the following: (1) Fabrication complexity remains the foremost barrier to scalable deployment. While SERS substrates achieve remarkable sensitivity through precisely engineered nanostructures (e.g., 2 nm gap Au dimers [107]), their production relies on costly e-beam lithography (~$500/chip [131]) with <60% yield. Similarly, MOF synthesis often requires 72 h solvothermal processes at 120 °C [115], consuming 30× more energy than nanoparticle production. These limitations restrict applications where disposable, mass-produced sensors are needed, such as pandemic screening or home testing. (2) Matrix interference severely compromises performance in real biological samples. Studies show that serum proteins reduce LSPR shift signals by 40–60% [108], while stool samples introduce >80% false positives in pathogen detection [132]. Current solutions like centrifugal filtration add complexity, increasing processing time from minutes to hours—negating the technology’s rapid response advantage. The lack of universal antifouling coatings forces case-by-case optimization, delaying clinical translation. (3) Scalability and cost remain hurdles, particularly for noble metal-based sensors and cleanroom-fabricated photonic devices [109,131]. (4) Standardization is lacking, with no universal protocols for performance validation [107,128].

Future directions may include the following: (1) Hybrid material systems show exceptional promise for overcoming stability–cost trade-offs; for example, MOF-encapsulated plasmonic NPs (e.g., ZIF-8@AuNRs [115]) combine 90-day environmental stability with 0.1 nM sensitivity, while 2D material heterostructures (graphene-hBN-MoS_2_ [112]) enable >100× reuse cycles while maintaining 90% initial sensitivity. Also, bioinspired designs (mussel foot protein coatings [108]) reduce biofouling by 75% without sample pretreatment. (2) Wearable integration is advancing, as demonstrated by the development of flexible hydrogel patches with embedded GMR sensors [120] that monitor chronic wound biomarkers (IL-6 and TNF-α) at 6 h intervals, contact lens platforms using glucose-responsive photonic crystals (5 min refresh rate [114]), and subdermal implants for continuous drug monitoring (in phase I trials for vancomycin detection [129]). (3) AI/ML co-design is revolutionizing two fronts: (1) fabrication optimization, with generative adversarial networks (GANs) predicting optimal nanostructures (e.g., 97% accuracy for SERS hot-spot design [124]), and (2) data interpretation, with convolutional neural networks decoding multiplexed SERS spectra with 95% accuracy vs. 68% for PCA [111], enabling 10-plex detection from single samples.

In summary, nanophotonic biosensors stand at a critical inflection point, transitioning from lab marvels to essential tools addressing global health and environmental challenges. Their unparalleled sensitivity (reaching single-molecule detection [105]) and multiplexing capacity (9-plex in <20 min [122]) already outperform those of gold-standard techniques across diagnostics, environmental monitoring, and food safety applications. However, as this analysis reveals, achieving widespread adoption demands coordinated advances: (1) manufacturing innovation, with the development of low-cost, high-yield nanofabrication methods to replace cleanroom dependence; (2) regulatory frameworks, with the establishment of ISO standards for performance validation and quality control; and (3) interdisciplinary collaboration, with the convergence of materials science, AI, and clinical research to address matrix challenges.

The coming decade will likely witness nanophotonics enabling the following: personalized medicine, with implantable sensors for real-time therapeutic drug monitoring; precision agriculture, with field-deployable phytopathogen detection at 1/100 of the current costs; and pandemic prevention, with airport-based pathogen screening with 90% sensitivity < 5 min. While challenges remain formidable, the coordinated efforts of academia, industry, and regulators can transform these technological triumphs into tangible societal benefits—ushering in an era where advanced diagnostics are as accessible as smartphone technology.

## 4. Nanophotonics in Medicine and Healthcare

Nanophotonics has become a field for innovation in medicine and healthcare. This permits us to design novel diagnostic and therapeutic tools that take advantage of nanomaterial characteristics and light–matter interactions. They are used to detect diseases in the early stages and develop non-invasive medical procedures [133,134]. However, the clinical use of nanophotonic applications in healthcare faces some challenges, for example, biological barriers, safety and toxicity concerns with nanoparticles, systemic obstacles, and a complex regulatory environment. The need for safer nanoprobes with few adverse side effects is continuously growing. This section discusses recent advances in the field of nanophotonic applications in medicine and healthcare, for example, the recent advances in photothermal therapy, applications for AI in nanophotonic healthcare devices, the role in detecting microRNA cancer markers, enhanced chemotherapy and imaging modalities, and image-guided surgery (IGS) enhanced by nanophotonics.

### 4.1. Nanophotonics for Photothermal Therapy of Tumors

**Figure 9:**
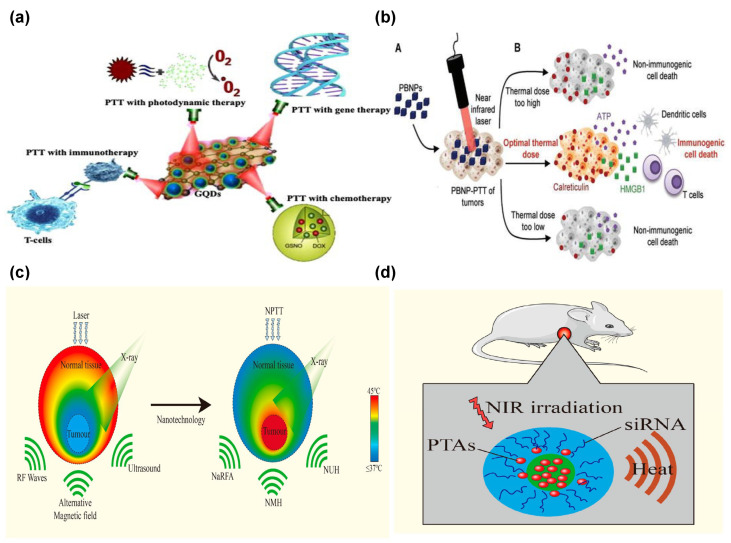
Nanophotonic approaches in medicine for photothermal therapy. (**a**) PTT in combination with different therapeutics [28]. (**b**) Illustration of how PTT causes immunogenic cell death [28]. (**c**) Combination of PTT with radiotherapy [151]. (**d**) Combination of PTT with gene therapy [151].

Photothermal therapy (PTT) is used to induce apoptosis and eliminate tumors through nanomaterials that absorb laser energy and convert it to localized heat [28]. It can target the tumor, reducing the damage to nearby healthy tissue. A prominent development in this area is the combination of PTT with other treatment modalities in order to generate synergistic anti-tumor effects. Employing nanostructures in PTT improves its effectiveness in cancer therapy [135]. Their nanoscale size enables passive accumulation in tumor tissues [136]. The distinctive optical characteristics of different nanomaterials, notably their high absorption in the near-infrared (NIR) region (700–1700 nm), are crucial for their application in PTT. The introduction of surface-modified NPs allows for selective administration, resulting in a carefully regulated rise in the local temperature (Figure 9) [137].

#### 4.1.1. Nanomaterials as Photothermal Agents (Ptas)

##### Noble Metal-Based Nanomaterials

Noble metal nanoparticles, which include substances such as gold, silver, platinum, and palladium, have received a lot of attention in PTT due to their significant surface plasmon resonance and ability to absorb light at specific near-infrared wavelengths. As a result, they may be used as excellent photosensitizers to facilitate photothermal conversion and increase efficiency. In this section, we focus on the use of noble metal nanoparticles, including gold, silver, platinum, and palladium, in the field of cancer [138].

The five main types of Au NPs used in PTT that have garnered the most interest throughout the preclinical development or clinical trial stages are Au nanoshells, Au nanorods, Au nanostars, Au nanocages, and Au nanospheres. Although size affects cellular uptake, with small NPs improving cellular absorption, shape also affects cellular uptake. For instance, Au nanorods (NRs) exhibit reduced cellular absorption compared to other Au nanostructures due to their aspect ratio [139]. Gold nanostars, with their sharp tips, can induce larger hot spots, leading to higher PCE compared to nanospheres or nanorods [140]. Gold nanoshells have been investigated in clinical trials and demonstrated efficiency in treating prostate cancer, being effective in 94% of cases when applied during ablative surgery, with 87.5% of biopsies being negative one year later [141]. On the other hand, spherical AuSHINs are frequently favored over gold nanorods due to their reduced toxicity and improved colloidal stability, which may require the use of cytotoxic surfactants during production [142]. Novel delivery methods such as nanostraw-assisted injection have been investigated, with results indicating approximately 10-fold higher internalization of gold shell-isolated nanoparticles (AuSHINs) and a 2-fold greater reduction in breast cancer cell viability compared to conventional incubation. A silver nanoprism has been shown to have considerable promise in photothermal treatment (PTT) due to its high surface plasmon resonance band in the near-infrared area. However, its instability under physicochemical conditions and extreme toxicity limit its future use [143].

Palladium nanoparticles have strong thermal and chemical stability, catalytic activity, and a tunable optical response [144]. Palladium is a key component in bimetallic nanoparticles (Ag-Pd NPs) stabilized by elm pod polysaccharide (EPP). Under near-infrared laser irradiation (808 nm), Pd-containing NPs reached 53.8 °C vs. 43.5 °C for Ag-only NPs [145]. Platinum was the first metal to be utilized in cancer therapy. Platinum nanoparticles (PtNPs) have been reported to exhibit anticancer activity and the ability to enhance anti-tumor therapy [146]. For instance, PEG@Pt/DOX is an integrated system with both therapeutic and diagnostic capabilities. These capabilities enable the use of computed tomography (CT) imaging along with the combined use of chemotherapy and photothermal treatments [147]. Hyaluronic acid (HA)-modified platinum nanoparticles (PtNPs) presented significant cytotoxicity toward the aggressive MDA-MB-231 cell line in vitro and inhibited tumor growth in vivo using PTT. This suggests that the targeting of HA-mediated and tumor-penetrating nanosystems can reasonably improve therapeutic performance in vivo. However, these platinum-based drugs have shown toxic effects on the kidney, brain, nerve tissue, and bone marrow, leading to side effects [148].

##### Carbon-Based Nanomaterials

Carbon-based nanomaterials (CBNs) have attracted a lot of interest as photothermal agents because of their particular optical, thermal, and chemical properties, as well as their large surface area and biocompatibility [149]. Carbon nanotubes (CNTs) exhibit great NIR absorption, high PCE, thermal conductivity, and potential for drug delivery. There are two types of CNTs: single-walled CNTs (SWCNTs) and multi-walled CNTs (MWCNTs), which have a variety of features. SWNTs are graphitic helical molecules with superior physical and mechanical characteristics. SWNTs are highly water-soluble, have low toxicity, and exhibit excellent biological stability. Adding polyethylene glycol (PEG) to SWNTs lengthens the blood circulation time. The introduction of Cy5.5 to SWNTs resulted in the development of Cy5.5-coupled SWNT-mediated PTT, and it achieved systemic tumor ablation in mice due to its absorption at 808 nm. Cy5.5 enhanced PTT’s therapeutic index.

Multi-walled carbon nanotubes (MWNTs), which range in diameter from a few nanometers to a few micrometers, have become popular options for biological imaging, photothermal tumor ablation, and tumor medication administration. MWNTs can produce localized heat upon NIR exposure, which can thermally annihilate tumors. Pathological analysis of the kidney, spleen, liver, and heart revealed that these modified CNTs were biocompatible and did not cause harm to any of the organs. These nano-agents have enormous promise in clinical and therapeutic applications, with relatively minor long-term safety concerns [149].

##### Graphene and Its Derivatives

Graphene and its derivatives are utilized in PTT for their biocompatibility, ease of production, tunable surface properties, and greater water solubility [150]. They exhibit significant NIR absorption, high PCE, a large surface area, and drug-loading capability. Carbon dots (CDs) are zero-dimensional nanocompounds known for their excellent optical properties, biocompatibility, and low cost and are used as fluorescent agents in imaging. CDs can produce synergistic regulatory cell death (RCD) pathways, such as necroptosis, using imaging-guided PTTs. For example, soya lecithin-coated red fluorescent carbon dot LRCDs show improved bioavailability and therapeutic properties in breast cancer. Table 1 summarize recent nanomaterials development for PTT.

**Table 1:** Summary of nanomaterials used in PPT and its features.

Nanomaterial Type	Key Properties for PTT	Examples
Carbon Nanotubes (CNTs)	Strong NIR absorption, high PCE, high thermal conductivity, and drug delivery potential	SWNTs MWNTs
Graphene and Derivatives	Exceptional NIR absorption, high PCE, large surface area, and drug-loading platform	Graphene Nanosheets Graphene Oxide (GO) Reduced Graphene Oxide (rGO) Graphene Quantum Dots (GQDs)
Carbon Dots (CDs)	Exceptional optical properties, biocompatibility, cost-effectiveness, and fluorescence for imaging	LRCDs

#### 4.1.2. Two-Dimensional (2D) Materials and Transition Metal Dichalcogenides (Tmds)

Molybdenum disulfide (MoS_2_) has photothermal absorption (PTA) characteristics. It is a CT imaging agent, has low toxicity, and is rapidly biodegradable. Also, it is an excellent NIR absorber with high optical stability and PCE [152]. MoS_2_-Ru nanocomposites exhibit synergistic CDT/PTT, a 60.9% apoptotic ratio in TNBC cells, a PCE of 41%, and superior catalytic activity (500 ng/mL compared to 20 µg/mL for MoS_2_ alone) [153]. Black phosphorus (BP) has unique tissue-responsive properties and is biocompatible and biodegradable. HA@BCN combines HA hydrogel with BCN and is used to treat cutaneous melanoma, where it has achieved >80% cancer cell mortality and tumor inhibition. Also, BP-PTX increases the permeability of cells to chemotherapy. The combination of BP nanosheets + R837 improves immune activity in PTT-driven cancer immunotherapy. Titanium carbide (Ti_3_C_2_ MXene) has many applications in bone regeneration and exhibits antibacterial activity. The combination of Ti_3_C_2_ + ferrous ions improves synergistic PTT–chemodynamic treatment. Also, Ti_3_C_2_ nanosheets exhibit concentration-dependent antibacterial activity. Niobium carbide (Nb_2_C) is used for deep tissue penetration in NIR-II PTT. Nb_2_C-PVP demonstrated deeper tissue penetration with a 1063 nm laser than with a 808 nm laser. Sharp Nb2C inhibits bacterial biofilm formation and increases heat sensitivity. Also, iron phosphoselenide (FePSe_3_) can be used in immune checkpoint therapy. For example, FePSe_3_ + APP caused an increase in antibody-labeled T-cell-related immunoreactions after PTT, which was mediated by blocking PD-1 [154]. Moreover, MXene-based nanomaterials have been utilized in multifunctional devices for upconversion, photothermal conversion, and enhanced luminescence [155,156,157,158].

#### 4.1.3. Organic and Other Inorganic Nanomaterials

Beyond noble metals, carbon-based materials, and TMDs, a wide range of additional organic and inorganic nanomaterials are being investigated for PTT, each with its own set of benefits and adding to the diversity of this therapeutic strategy. Organic nanomaterials have higher biocompatibility and biodegradability than many inorganic equivalents, which is critical for reducing long-term toxicity and enabling removal from the body. Polydopamine (PDA) is a commonly utilized component or coating for nanoparticles. It is made by oxidizing dopamine in situ. It has good light absorption and high PCE, making it a popular organic material for PTT [159]. Indocyanine green (ICG), an FDA-approved photosensitizer used for clinical diagnostics, can cause hyperthermia and induce the production of reactive oxygen species (ROS) when exposed to NIR lasers. Because of its low stability and fast removal in its original form, ICG is commonly encapsulated into different nanocarriers to increase its bioavailability and therapeutic potential. Examples are mPEG-luteolin-BTZ@ICG for colorectal cancer and ICG-lactosomes for breast cancer. Cypate is a bis-carboxyl-containing indocyanine green derivative that emits fluorescence and produces heat when exposed to NIR light. Cyp-PMMA-Fe@MSCs have been investigated for the diagnosis of lung cancer.

IR-780 is a hydrophobic heptamethine dye that emits more fluorescence than ICG, but it is less soluble and quickly removed. Encapsulation in amphiphilic micelle nanoparticles improves stability. Conducting polymers, such as polyaniline (PANI) and polypyrrole (PPy), are commonly employed for their high optical absorbance, low cost, and biocompatibility. PANI was one of the first documented polymer-based photothermal anticancer drugs, whereas PPy has a high PCE and improved biocompatibility. Melanin-like polymers are found in plants, animals, and people and have antioxidant, radioresistant, and anti-neoplastic characteristics, as well as strong NIR radiation absorption. Melanin PEGylated nanoliposomes have shown promise for therapeutic use in skin cancer. Naphthalocyanines and phthalocyanines are chemical dyes designed for strong NIR absorption, reducing light scattering and absorption by biological tissues. Phthalocyanine, for instance, is a well-known organic PTA with a well-defined chemical structure that can be synthesized repeatedly [149].

On the other hand, inorganic materials such as semiconductor materials exhibit excellent NIR absorption and PCE. These include 2D materials like copper, bismuth, and tungsten chalcogenides (e.g., CuS and Bi_2_S_3_). The characteristics of long-term biocompatibility and degradation patterns are still being investigated. Although iron (Fe)- and manganese (Mn)-based oxides have lower intrinsic PCE, they are widely studied. They have multipurpose potential, like application as MRI contrast agents or magnetic hyperthermia effectors. Iron oxide nanoparticles (IONPs) are degraded by lysosomes into Fe ions, and they are eliminated through the metabolic pathway [160].

#### 4.1.4. Ptt in Combination with Conventional Therapies (Chemotherapy, Radiotherapy, and Gene Therapy)

PTT + chemotherapy improves drug delivery, triggers drug release, overcomes drug resistance, and increases cell membrane permeability. For example, PEG@Pt/DOX and PTX-GO-PEG-OSA improve cytotoxic effects on drug-resistant breast cancer and gastric cancer cells. ZrC nanosheets enhance tumor inhibition through prodrug activation and release of chemotherapeutic medicines upon laser irradiation. PTT + radiotherapy acts by inhibiting DNA repair, synchronizing cell cycles, increasing oxidative stress, overcoming radioresistance, and killing complementary cells. For example, mild hyperthermia (41–43 °C) improves radiosensitivity with a thermal enhancement ratio of (1.5–5.7) [151]. In addition, W_18_O_49_ nanospheres have excellent radiation sensitization and photothermal performance, suppressing tumor proliferation and metastasis. PTT + gene therapy enhances tumor cell apoptosis and improves the delivery of genetic material. In addition, MSC membrane-camouflaged PDA cores enable synergistic chemo-photothermal therapy and gene therapy by delivering siRNA.

#### 4.1.5. Ptt Synergies with Immunotherapy

This combination enhances mechanisms like immunogenic cell death (ICD), tumor microenvironment (TME) modulation, immune cell infiltration, and synergy with immune checkpoint blockade (anti-CTLA-4 and anti-PD-L1/PD-1). For example, combining metal-based nanomaterials (Au, Ag, Pt, Pd, and TMDCs) with anti-CTLA-4 and anti-PD-L1/PD-1 therapy enhances their anticancer activity [161]. In addition, the combination of corn-like Au/Ag nanorods + anti-CTLA-4 induces immune memory and prevents tumor recurrence [162]. Also, when nanodrugs are directly or indirectly targeted to the tumor site and release tumor-associated antigens and cell fragments, PTT can cause immunogenic cell death, which activates systemic immunity and eradicates any remaining or metastatic malignancies [28]. Finally, the combination of FePSe3 nanosheets + anti-PD-1 prolongs survival by promoting dendritic cell maturation and T-cell activation.

#### 4.1.6. Ptt Integration with Other Light-Activated and Emerging Therapies (Pdt, Cdt, and Sdt)

The combination of PTT + photodynamic therapy (PDT) exerts synergistic effects by alleviating hypoxia, producing ROS, and employing complementary cell death mechanisms. For example, MWCNT-mTHPC, which is a combination of PDT and PTT, induces apoptosis through oxidative stress-mediated and mitochondrial damage in ovarian cancer. In addition, graphene (GFN) enhances ROS formation in both PDT and PTT. Finally, covalent organic framework nanoparticles increase the PTT temperature, thereby significantly enhancing PDT sensitivity.

The combination of PTT + chemodynamic therapy (CDT) achieves synergistic ROS production via Fenton-like reactions, specifically targeting the TME. In addition, MoS2-Ru nanocomposites, which enhance catalytic activity and photothermal conversion, lead to synergistic CDT/PTT outcomes in TNBC cells. Finally, Ti_3_C_2_ nanosheets and ferrous ions provide synergistic PTT–chemodynamic treatment by generating ROS.

The combination of PTT + sonodynamic therapy (SDT) involves combined activation by light and ultrasound, which enhances ROS generation in deep tumors. In addition, the CuS-Pt complex improves photothermal performance and catalyzes O_2_ generation, which improves synergistic tumor killing.

### 4.2. Applications for AI in Nanophotonic Healthcare Devices

AI integration with nanophotonic healthcare devices has achieved major advances in diagnosis and treatments using advanced light engineering at the nanoscale with biomolecules.

#### 4.2.1. AI-Enhanced Nanophotonic Diagnostics

Nanophotonic biosensors can be used for the early diagnosis of complex diseases like cancer [163]. They have the ability and sensitivity to detect biomolecules in the absence of labels [8]. AI can improve the processing of optical signals, thereby increasing biomarker true sensitivity, specificity, and accuracy [164]. It can identify small shifts in spectra or changes in intensity within SPR sensors. This can lead to the detection of small amounts of target analytes [165]. In addition, AI can provide many datasets that can help researchers in many disease scenarios [166]. During the COVID-19 pandemic, the integration of AI nanophotonic biosensors aided in the rapid evaluation of the presence of infection and the early detection of the virus [112].

AI has been integrated in nanophotonics. Imaging modalities like OCT provide high-resolution images of biological structures [167]. Deep learning models enhance imaging technologies by improving resolution and reducing image noise, thus facilitating accurate diagnosis [168]. In addition, AI can transform images from one modality to another, thereby allowing relevant information to be extracted from one imaging modality using data from another [169].

#### 4.2.2. AI-Driven Nanophotonic Therapeutics

**Figure 10:**
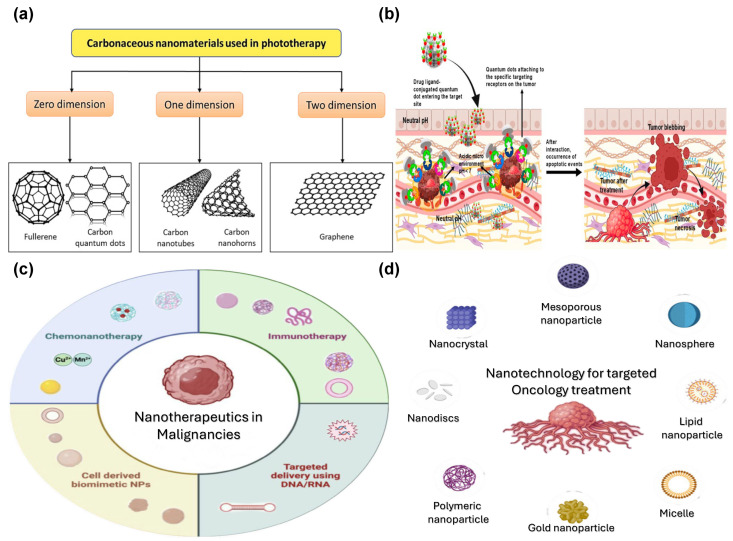
Nanophotonic approaches in healthcare for diagnostics and treatment. (**a**) Dimensions of nanomaterials, particularly carbonaceous nanomaterials [187]. (**b**) Illustration of how drug–ligand-conjugated QDs deliver chemotherapeutic medicines with a specific activity [29]. (**c**) Nanotherapeutic strategies in malignancies [188]. (**d**) Nanotechnology for targeted oncology treatment [188].

Nanophotonics may be used in drug delivery. AI can enhance the targeting capabilities of nanoparticles and improve patient treatment outcomes [170]. AI can improve the delivery of drugs by predicting the nanoparticle shape required for maximum distribution within tumor tissues [171]. Nanophotonics can also play a role in therapeutic platforms [172]. AI can be used to optimize the nanophotonic agents used in PTT and PDT by determining whether to capture more light at particular therapeutic wavelengths, improve heat generation, or increase ROS production, which improves the efficacy of these light therapies (Figure 10) [173].

Nanocarriers are a system used for the delivery of drugs or therapeutic genes and small interfering RNA molecules [174]. When using gene delivery nanocarriers, AI technology is primarily depended on to optimize biological stability, promote cellular entry, and enhance target cell recognition. Leveraging the analytical capabilities of AI improves the outcomes of gene therapy [175].

### 4.3. The Role of Nanophotonics in Detecting MicroRNA Cancer Markers

Surface plasmon resonance (SPR) and localized surface plasmon resonance (LSPR) sensors are optical sensing technologies that detect minor changes in the local refractive index to monitor a molecule’s attachment to a metal surface [176]. Exciting polarized surface plasmons (SPPs) generate an evanescent wave to detect binding events occurring at the surface [177]. Localized surface plasmon resonance (LSPR) is a type of plasmonic sensing that is different from SPR sensing; it does not require prism coupling and allows for simpler and smaller optical systems. The range of LSPR could detect miRNAs at very low concentrations, typically molar amounts, when combined with methods that amplify their signals [178,179].

Photonic crystals (PCs) are periodical nanostructures that are manufactured to control the transmission of light and form optical bandgaps [180]. Using photonic crystal surfaces increases the excitation of fluorescently tagged biomolecules and efficiently channels photons to detectors [181]. The combination of photonic crystal arrays and molecular amplification techniques such as CRISPR/Cas12a and HCR can greatly improve the sensitivity of detection for low-abundance miRNAs. More complex designs, such as encoding oriented conformal resonance (GMR) sensors, facilitate overall system design and allow for simultaneous measurements of many analytes, as shown in [182]. Photonic crystal sensors allow for both optical and molecular signals to be amplified, making them truly remarkable.

Quantum dots (QDs) are semiconductor nanocrystals with excellent optoelectronic properties. They can make excellent fluorescent probes for miRNA detection, and their use is based on specific miRNA binding events [183]. A powerful technique used to monitor miRNA in QDs is called FRET [184].

### 4.4. Nanophotonic-Enhanced Chemotherapy

Plasmonic nanoparticles (e.g., gold and silver) are used in localized surface plasmon resonance (LSPR), and they are effective in photothermal conversion, have good photostability, and have reduced cytotoxicity. In chemotherapy, they improve drug delivery through light-to-heat conversion (PTT). They are also used in tumor ablation, drug injection, and bioimaging contrast improvement [185]. QDs are small in size and exhibit adjustable photoluminescence, a narrow emission, a high quantum yield, and photobleaching resistance [186]. In chemotherapy, they are used in drug delivery for ROS generation (PDT), bioimaging, photosensitizers, and real-time drug route monitoring [29]. Carbon-based nanomaterials (e.g., graphene, carbon dots, and nanodiamonds) exhibit a large surface area, mechanical strength, electrical/thermal conductivity, and tunable surface chemistry, and some are intrinsic photosensitizers. In chemotherapy, they are used for drug delivery through light-to-heat conversion (PTT), ROS generation (PDT), or drug encapsulation [187]. Liposomes are biocompatible, biodegradable, and temperature-sensitive, and they allow for drug encapsulation and sustained release. They are used for the delivery of chemotherapeutic medications (i.e., Doxil^®^) and light-triggered drug release (if they are temperature-sensitive). Polymeric nanoparticles are synthetic polymers used to ensure drug safety, control drug release, and target drug delivery (EPR/active); for example, paclitaxel/doxorubicin encapsulation is used for active targeting [188,189].

### 4.5. Imaging Modalities and Image-Guided Surgery (Igs) Enhanced by Nanophotonics

For most solid tumors, surgical excision is still the most common and successful therapeutic option [190]. However, reducing the likelihood of tumor recurrence and enhancing long-term survival depend on obtaining total tumor excision with negative surgical margins or there being no malignant tissue left at the corners of the removed specimen. The sensitivity and specificity required to detect microscopic residual disease or precisely define tumor borders in real time are sometimes lacking in the conventional techniques used to evaluate surgical margins, such as intraoperative frozen section analysis (IFSA). Positive surgical margins (PSMs), which require subsequent treatments or reoperations and expose patients to extra side effects and financial stress, are often the result of this [191]. Image-guided surgery (IGS) seeks to overcome these significant restrictions by providing surgeons with real-time optical imaging feedback during the process. IGS is defined here as surgery guided by real-time optical imaging feedback. The use of nanophotonics has greatly improved numerous essential imaging modalities, increasing their usefulness in minimally invasive and image-guided surgical operations. These advancements are discussed in this section.

#### 4.5.1. Fluorescence-Guided Surgery (FGS)

FGS is a popular image-guided surgical technology that gives surgeons real-time visual feedback during procedures. Although fluorescent image-guided surgery (FIGS) offers significant benefits over traditional imaging techniques, its limitations include shallow tissue penetration and autofluorescence from clinically approved fluorophores. To address this issue, researchers are moving from visible to longer wavelengths. The NIR spectrum, which includes NIR-I (700–900 nm) and NIR-II (950–1700 nm), increases signal-to-noise ratios and enables deeper tissue penetration. Recent advancements in nanophotonics have focused on nanoformulations of NIR-II fluorophores to increase in vivo stability and tumor-targeting delivery. Soft NPs with NIR-II fluorophores are widely employed as contrast agents. PDFT1032 polymeric NPs, for example, are composed of PDFT, a novel NIR-II-emitting polymer, wrapped in a DSPE-mPEG shell. PDFT1032 induced high TBRs in osteosarcoma for up to 3 days after an IV injection [191].

#### 4.5.2. Photoacoustic Imaging (PAI)

PAI can be used in direct medication administration, treatment planning, and surgery. It can be used to assess intraoperative dense decalcified and non-decalcified bone samples. This technique might help identify intraoperative tumor margins and detect bone tissue disorders. In addition, multispectral optoacoustic tomography (MSOT), a type of PAI, has shown promise for SLN mapping when combined with indocyanine green (ICG). This method helps to rule out metastases. In patients with Crohn’s disease, the MSOT technique can be used to differentiate between intestinal remission and active disease, determine hemoglobin levels, and identify markers of inflammation. Nanoparticles can be used as contrast agents in image-guided surgery.

#### 4.5.3. Surface-Enhanced Raman Spectroscopy (SERS)

Recently, SERS has been employed in image-guided cancer surgery. Stimulated Raman scattering microscopy has enabled the imaging and detection of tumors that invade the brain. It has made it simpler to collect diagnostic histological data during brain tumor surgery. It has also demonstrated potential at the preclinical stage for distinguishing tissues after tumor surgery [192].

#### 4.5.4. Optical Coherence Tomography (OCT)

OCT provides high-resolution imaging and is non-invasive [193]. It provides detailed anatomical structure information. An important advantage is that it provides label-free molecular contrast. OCT has been widely used in different fields like cardiovascular disease, ophthalmology, and dermatology. It can identify malignancies in a variety of tissues. For example, it helps identify brain tumors. In addition, it can be used for tissue differentiation and margin assessment. As another example, optical coherence elastography (OCE) has made it possible to identify any remaining cancer cells in the surgical cavity after breast-conserving surgery (BCS) in vivo.

#### 4.5.5. Upconversion Nanoparticles (UCNPs)

The goal of developing UCNPs is to improve bioimaging. They are used as a tool in image-guided treatments and diagnostics, for example, to detect cancer, such as lymph node metastases. Detecting lymph node metastases is an important step in cancer staging and treatment planning. They are also combined with theranostic platforms, which aim to enable simultaneous fluorescence imaging and chemo-photodynamic combination treatment [193].

#### 4.5.6. Quantum Dots (QDs)

QDs are employed in fluorescence-guided cancer surgery and diagnostics. They are also utilized in vivo in molecular and cellular imaging [64]. In vivo studies have also examined their potential for lymph node imaging. Some varieties, such as PbS quantum dots, provide fluorescence imaging for breast tumors [190].

### 4.6. Critical Challenges and Clinical Translation of Nanophotonics in Medicine

Although nanophotonics is rapidly growing, there is still a large gap between lab experiments and real hospital application. Most nanophotonic tools work well in a controlled lab, but they often fail in the complex environment of a real clinic. Even though these systems are very sensitive, their performance depends too much on ideal conditions that are hard to achieve in everyday medical practice. In diagnostics, for example, many devices show amazing results in tests. However, when they are used with real biological samples, things get complicated. Problems like “noise” from the body or differences between patients make it hard to trust these sensors every time. Also, producing these devices on a large scale is tough. Small mistakes during manufacturing mean that one device might work differently from the next, which stops them from receiving official medical approval. Imaging tools, like super-resolution systems, also face hurdles. They give very clear pictures, but the equipment is often too complex and fragile. They need constant fixing and perfect alignment, which most hospitals cannot afford or manage. This means that only a few specialized centers can use them, while most patients cannot. Therapies like photothermal treatment also have serious issues. We still do not know enough about how nanoparticles move through the body or whether they are toxic in the long term. It is also hard to control the “dose” of light and heat accurately, which might accidentally damage healthy tissue. Additionally, large-scale fabrication of such nanomaterials remains costly and time-consuming. Therefore, for nanophotonics to truly help patients, we need to stop just looking for “high performance.” Instead, we should focus on making tools that are reliable, cheap, and easy to manufacture. The future of this field depends on simple, practical tools that actually work in a doctor’s hand, not just in a researcher’s lab.

## 5. Nanophotonics for Artificial Intelligence and Optical Computing

The exponential growth of data-centric applications and the escalating computational demands of artificial intelligence (AI), particularly deep learning, have exposed fundamental limitations in conventional electronic computing architectures rooted in the von Neumann paradigm. The physical separation of memory and processing units creates an intrinsic bottleneck in data transfer, consuming excessive energy and constraining processing speeds, especially for the matrix multiplications and convolutions inherent in neural network operations. Nanophotonics, which explores light–matter interactions at subwavelength scales, offers a revolutionary pathway to transcend these limitations. By harnessing photons as information carriers, nanophotonic systems promise ultra-high bandwidth, massively parallel processing capabilities, minimal heat dissipation, and the potential for direct manipulation of optical information (e.g., images and sensor data) without inefficient electro-optical conversions [194,195,196,197]. This section comprehensively examines the cutting-edge advancements in nanophotonics for AI and computing, delving into the underlying principles, diverse architectures, novel materials, and key devices driving progress in optical neural networks (ONNs), neuromorphic photonic computing, and emerging quantum photonic approaches. A critical analysis of the persistent challenges and limitations confronting the field is also presented, alongside insights into ongoing research strategies aimed at realizing the transformative potential of light-based intelligent computing systems.

### 5.1. Optical Neural Networks (ONNs)

Optical neural networks (ONNs) represent a direct physical implementation of artificial neural network architectures using photonic components, capitalizing on the inherent parallelism, speed of light propagation, and energy efficiency of optical systems to perform core computational tasks in AI, such as classification, regression, and feature extraction. Nanophotonics provides the essential toolkit for miniaturizing and integrating the fundamental building blocks—artificial neurons and synapses—and efficiently interconnecting them on-chip or in free space.

#### 5.1.1. Core Architectures and Platforms

ONNs are primarily categorized based on their physical implementation platform. Free-space optical neural networks (FSONNs) utilize bulk optics or planar optical elements arranged in three-dimensional space to manipulate light waves representing data. Central to FSONNs are diffractive optical elements (DOEs), particularly multilayer diffractive structures known as diffractive deep neural networks (D^2^NNs) and metasurfaces, where light propagation between layers performs the linear transformations (matrix multiplications) fundamental to neural networks via diffraction and interference. Montes McNeil et al. provide a detailed taxonomy of FSONNs, highlighting implementations based on 3D-printed layers, dielectric or plasmonic metasurfaces, and spatial light modulators (SLMs) [198]. FSONNs excel in massive parallelism and direct handling of large input datasets like full images but often grapple with challenges in reconfigurability, sensitivity to alignment, and large physical footprints. Significant progress was made through pluggable multitask diffractive neural networks employing cascaded metasurfaces [199,200,201]. By fixing one metasurface and switching pluggable metasurface modules, their system was reconfigured to perform distinct recognition tasks, for example, handwritten digits versus fashion products at near-infrared wavelengths, exemplifying a pathway toward versatile, high-speed, and low-power multifunctional AI systems. Similarly, Tang et al. introduced an “Optical Neural Engine” (ONE) architecture that synergistically combines diffractive networks for Fourier space processing with optical crossbar structures for real space processing, enabling efficient and reconfigurable solutions to complex scientific partial differential equations (PDEs) [202].

**Figure 11:**
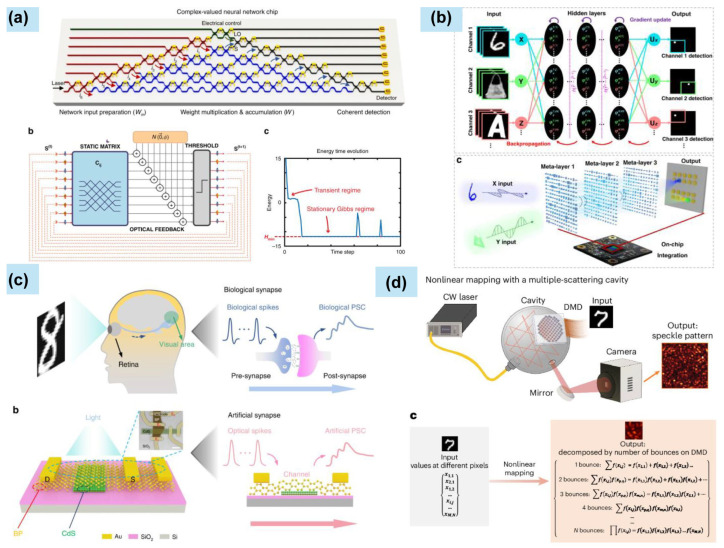
Comparative architectures for AI computing. (**a**) Integrated photonic neural network (IPNN) core performing in-memory matrix multiplication using a Mach–Zehnder Interferometer (MZI) mesh or bank of microring resonators (MRRs). Reprinted with permission from Springer Nature [207]. (**b**) Free-space optical neural network (FSONN) employing cascaded diffractive layers (e.g., metasurfaces) for massively parallel optical computation. Reprinted with permission from Springer Nature [208]. (**c**) Neuromorphic photonic system featuring spiking signals generated by nano-optoelectronic neurons (e.g., RTD-based) communicated via optical interconnects and processed by memristive synaptic crossbars. Reprinted with permission from Springer Nature [209]. (**d**) Nonlinear optical neural networks featuring an optical cavity with multiple internal reflection. Reprinted with permission from Springer Nature [210].

In contrast, integrated photonic neural networks (IPNNs) confine and guide light within nanoscale waveguides fabricated on semiconductor substrates like silicon or silicon nitride. Key components include Mach–Zehnder Interferometers (MZIs) and microring resonators (MRRs) (Figure 11). MZI meshes can be programmed to implement arbitrary unitary matrices, performing matrix multiplications, while MRRs modulate light intensity through resonance shifts, acting as tunable weights or activation functions. Dong et al. extensively chronicle the evolution from free-space to on-chip platforms, emphasizing the roles of MZIs, MRRs, and metasurface-based diffractive networks within photonic circuits [203]. IPNNs offer advantages in compactness, stability, potential for co-integration with electronics, and high operational speeds. However, scalability remains a critical hurdle due to waveguide crossing losses, signal attenuation, and the relatively large footprint of tunable elements like MZIs. Gu et al. addressed this with “Squeeze Light,” a scalable ONN architecture utilizing multi-operand ring resonators (MORRs) to execute vector dot products within a single device, significantly enhancing compactness and efficiency compared to conventional MZI-based designs [204]. Qu et al. showcased an inverse-designed integrated nanophotonic ONN based on optical scattering units, achieving high-precision stochastic matrix multiplication and image classification (MNIST) within an ultra-compact footprint (4 × 4 µm^2^ per unit) [205]. Further blurring the lines between guided-wave and free-space approaches, Wang et al. explored the integration of metasurfaces directly onto silicon photonic platforms, creating on-chip analogues of lenses and spatial light modulators for enhanced signal processing and computing functionalities [206].

#### 5.1.2. Enabling Multifunctionality and Efficiency with Metasurfaces

Metasurfaces, planar arrays of subwavelength nanostructures (meta-atoms) that impart designed local phase, amplitude, and/or polarization shifts on incident light, have become a cornerstone technology for ONNs. Their subwavelength thickness enables ultra-compact devices and provides unprecedented control over light wavefronts. Within free-space D^2^NNs, metasurfaces are ideal for implementing diffractive layers. By spatially varying the meta-atom design, the precise phase profile required for specific diffraction patterns, representing desired linear transformations, can be encoded. Cheng et al. demonstrated such metasurface-based D^2^NNs for image classification tasks (handwritten digits and animals) using inverse-designed silicon nanodisks, achieving high optical transmittance and classification accuracy (>90% for digits) [211]. Luo et al. advanced the field with a multi-task optoelectronic hybrid neural network employing a nonlinear metasurface featuring U-shaped resonant units. This design generated second harmonic light, enabling phase multiplexing at both fundamental and second harmonic frequencies, which allowed simultaneous handwritten digit classification (95.53% accuracy) and image reconstruction on the MNIST dataset within a unified architecture [212].

Moving beyond static implementations, programmable and reconfigurable metasurfaces are crucial for adaptive learning and multifunctional systems. Wang et al. demonstrated a multichannel meta-imager utilizing an electrically tunable liquid crystal (LC)-integrated metasurface. By exploiting polarization and angle multiplexing dynamically controlled by applied voltages, the system exponentially increased the number of convolution kernels, performing both positive and negative convolutions simultaneously and achieving high accuracy in image classification (98.5% for digits and 90.9% for fashion images) [213]. Ma et al. conceptualized “Information Metasurfaces” and “Intelligent Metasurfaces,” merging the digital coding/programmable metasurface paradigm with AI for self-adaptive devices, intelligent imagers, and programmable optical AI machines [214]. The integration of metasurfaces with planar photonics is rapidly progressing. Wang et al. discussed their deployment on integrated photonic platforms as mode converters and novel light manipulation elements, offering functionalities beyond conventional waveguide devices for computation, imaging, and beam steering [206].

#### 5.1.3. Implementing Linear and Nonlinear Operations

The computational core of neural networks involves linear transformations (matrix multiplications) followed by nonlinear activation functions, both of which must be efficiently realized in ONNs. Linear operations are a natural strength of photonics. Both FSONNs (leveraging diffraction) and IPNNs (using MZI meshes, MRR banks, or scattering units) inherently excel at parallel matrix–vector and matrix–matrix multiplication due to the wave nature of light. Sludds et al. proposed a scalable coherent ONN architecture based on balanced homodyne detection, targeting scalability to millions of neurons with potential sub-fJ/MAC energy consumption, ultimately bounded by photodetector shot noise [215]. Hattori et al. implemented optical vector–matrix multiplication (VMM) circuits using wavelength division multiplexing (WDM) for ultra-wideband operation [216].

Introducing efficient optical nonlinearity, however, remains a significant challenge, crucial for enabling the complex decision boundaries in deep learning. Current strategies primarily involve optoelectronic or all-optical approaches. The optoelectronic method converts optical signals to electrical currents via photodetectors, applies electronic nonlinearity (e.g., using transistors), and then modulates light back using optical modulators. This hybrid approach offers flexibility but incurs latency and energy penalties from optical-to-electrical and electrical-to-optical (O/E/O) conversions. Pursuing all-optical nonlinearity seeks to leverage intrinsic material nonlinearities (e.g., χ^(3)^ effects like the Kerr nonlinearity in silicon and saturable absorption) or engineered structures (e.g., resonators enhancing nonlinear effects and phase-change materials). Aggarwal et al. demonstrated ultrafast switching in antimony (Sb) thin films, suggesting potential for nonlinear elements [217]. Chen et al. showcased ultrafast (2 ns) non-volatile photonic memory using Sc-doped Sb_2_Te_3_ (SST) phase-change material, adaptable for nonlinear responses [218]. Several groups consistently identify achieving efficient, low-power, and fast all-optical nonlinearity as a critical frontier for advancing ONNs, potentially involving nanolasers or other active elements as nonlinear neuron sources [203,219,220,221].

### 5.2. Neuromorphic Photonic Computing

Neuromorphic computing aims to emulate the structure (neurons and synapses) and event-driven, low-power information processing principles of biological neural systems. Neuromorphic photonic computing harnesses light to implement these bioinspired paradigms, offering compelling advantages in speed, bandwidth, and energy efficiency, particularly for spiking neural networks (SNNs) and related models.

#### 5.2.1. Photonic Memristive Synapses and Neurons

Memristors, resistive switching devices possessing inherent memory, are ideal candidates for artificial synapses due to their ability to store synaptic weights as conductance states and implement synaptic plasticity rules. Research has explored diverse nanoscale material systems for neuromorphic photonics. Oxide-based materials (e.g., HfO_2_ and TaO_x_) are prevalent in CMOS-compatible resistive RAM (RRAM). Mikhaylov et al. summarized CMOS-integrated memristive arrays (RRAM) for neuromorphic computing, emphasizing their use in crossbar arrays for efficient vector–matrix multiplication and the potential for orders-of-magnitude gains in performance and energy efficiency over traditional hardware [222]. Gayakvad et al. focused on spinel ferrites (e.g., CoFe_2_O_4_ and NiFe_2_O_4_) synthesized via spin coating for RRAM, discussing their resistive switching properties, endurance, and suitability for neuromorphic computing and hardware security applications [223]. Phase-change materials (PCMs) like Ge_2_Sb_2_Te_5_ (GST) and Sc-doped Sb_2_Te_3_ (SST) offer non-volatile, multi-level switching. Chen et al. demonstrated ultrafast neuromorphic photonic memory using SST with 2 ns write/erase speeds, showcasing multilevel capability, stability, and application as synapses in an ANN for image classification, alongside potential for reflective nanodisplays [218]. Two-dimensional materials (graphene and transition metal dichalcogenides—TMDCs) and nanostructures offer unique electronic and optical properties. Sun et al. reviewed synaptic devices (memristors and transistors) based on various nanomaterials (quantum dots, nanowires, 2D materials, oxides, ferroelectrics, and organics) for neuromorphic computing [224]. Panes-Ruiz et al. specifically reviewed carbon nanomaterial-based memristive devices (fullerenes, carbon nanotubes, and graphene) for neuromorphic applications [225]. Hassanzadeh discussed the broader potential of 2D nanoelectronic materials in bioinspired computing, including neuromorphic functions [226]. Perovskites represent another promising class; Ma et al. highlighted their use in optoelectronic synapses in addition to demonstrating CsPbBr_3_ nanoplate-based synapses emulating essential functions like short-term/long-term plasticity and learning-experience behavior, even featuring a unique memory backtracking capability [227,228].

The operation of photonic memristive devices involves setting the conductance state (synaptic weight) using electrical pulses, optical pulses, or a combination of stimuli (heterostimuli). Kim et al. exemplified heterostimuli chemo-modulation in ZnO/polyvinylpyrrolidone nanocomposite memristors, combining electrical switching with photostimuli-modulated redox chemistry for associative learning. This device exhibited rapid learning (1 ms) and good retention and, when integrated into an ANN crossbar, enabled highly data-efficient machine learning with exceptional power efficiency [229]. Sun et al. positioned memristor-based artificial chips as core components of future brain-inspired AI systems due to their innate in-memory computing capability, tracing their evolution from synapses to neural networks and brain-like chips [230]. Luan et al. emphasized the reciprocal development between ANNs and nanophotonics, where neural network algorithms like inverse design facilitate the creation of novel nanophotonic neuromorphic devices [219].

#### 5.2.2. Spiking Neural Networks and Event-Driven Processing

Spiking neural networks (SNNs) process information based on the precise timing of discrete spikes (events), closely mimicking biological neural communication and offering high energy efficiency for sparse data. Implementing photonic SNNs necessitates artificial spiking neurons and efficient optical spike communication channels. Emulating spiking neurons leverages various nanophotonic approaches. Nano-opto-electronic devices, particularly resonant tunneling diodes (RTDs) exhibiting folded negative differential resistance (NDR), enable intrinsic spiking behavior. Romeira et al. and Jacob et al. are pioneering brain-inspired nanophotonic spike-based devices using III-V nanoRTDs as high-speed artificial neurons [231,232,233]. Integrating these nanoRTDs with nanoscale light-emitting diodes (nanoLEDs) or nanolasers creates spiking emitter nodes, while integration with nanoscale photodetectors (nanoPDs) forms spiking receiver nodes. Semiconductor lasers subjected to optical feedback exhibit complex dynamics, including excitable (neuron-like spiking) and chaotic regimes, useful for reservoir computing and potentially direct spiking emulation. Wu et al. reviewed intelligent optical computing based on laser cavities, covering dynamics relevant to SNNs. Other active devices like VCSELs and microlasers with thresholding and relaxation oscillations can also be engineered for spiking [234].

Efficiently routing optical spikes between neurons is critical. Romeira et al. discuss employing silicon photonics interconnects, integrated photorefractive interconnects, and 3D polymeric waveguide interconnections. Synchronization of photonic spiking neurons, essential for coordinated computation, is an active research area, often exploiting the nonlinear dynamics and coupling between devices. Li et al. reviewed spin-wave excitation and synchronization in spin Hall nano-oscillators (SHNOs), highlighting their potential as nanoscale spiking signal sources for magnonic neuromorphic systems [235].

#### 5.2.3. Photonic Reservoir Computing

**Figure 12:**
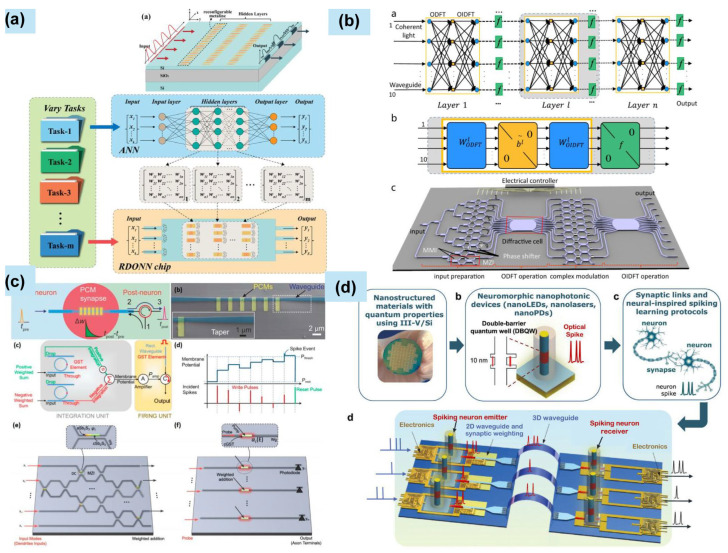
Key nanophotonic building blocks for artificial intelligence. (**a**) Integrated phase-change metasurface enabling precise wavefront control for D^2^NNs or on-chip light manipulation. Reprinted with permission from Optica [237]. (**b**) Essential integrated photonic components: Mach–Zehnder Interferometer (MZI) for linear algebra, microring resonator (MRR) for weighting/filtering, and the challenge of waveguide crossings. Reprinted with permission from Springer [30]. (**c**) Phase-change material (PCM) cell acting as a non-volatile photonic memory element or synaptic weight. Reprinted with permission from MDPI [238]. (**d**) Nano-opto-electronic spiking neuron concept integrating a resonant tunneling diode (RTD) with a nano-light-emitting diode (nanoLED) or nano-photodetector (nanoPD) for optical spike generation/detection. Reprinted with permission from IOP [233].

Reservoir computing (RC) is a neuromorphic paradigm utilizing a fixed, randomly connected recurrent network (the “reservoir”) with nonlinear nodes to transform input signals into a high-dimensional state space. Only a simple readout layer, typically linear, requires training, simplifying learning for temporal tasks. Photonics is well-suited for implementing the reservoir due to inherent dynamics and parallelism. Common implementations involve delayed feedback systems, where a single nonlinear node (e.g., a semiconductor laser, modulator, or photodetector) with a delayed feedback loop creates virtual nodes in time. Brunner et al. pioneered this using a semiconductor laser, with Wu et al. reviewing subsequent advances employing various lasers (semiconductor ring lasers, microchip lasers, and VCSELs) and photonic integrated circuits [234]. Spatial photonic reservoirs consist of networks of coupled photonic nodes (e.g., microring resonators and photonic crystal cavities) implemented on-chip. Dan et al. included RC within broader discussions of intelligent photonic and analog optical computing. RC excels in applications like time-series prediction, speech recognition, and chaotic system modeling, benefiting significantly from the high speed and bandwidth of photonic realizations (Figure 12) [236].

### 5.3. Quantum Photonics for AI Applications

While classical nanophotonics underpins current AI hardware research, quantum photonics leverages quantum mechanical phenomena (superposition and entanglement) and holds long-term promise for specific AI applications demanding exponential speedup or processing inherently quantum data. This field remains nascent regarding direct nanophotonic hardware for mainstream AI. Quantum neural networks (QNNs) are theoretical models proposing the use of quantum systems (entangled photons and superconducting circuits) to represent and process information, potentially accelerating certain machine learning algorithms. Implementing QNNs necessitates stable, scalable quantum photonic hardware: integrated sources of entangled photons, low-loss reconfigurable quantum photonic circuits (using components like MZIs and phase shifters), and efficient single-photon detectors. Progress in integrated quantum photonics forms the essential foundation, though specific nanophotonic AI hardware demonstrations are limited. Concepts from quantum computing, like quantum walks or Ising models, can sometimes be mapped onto classical photonic hardware for potential acceleration in optimization and sampling tasks. Wu et al. discussed photonic Ising machines using injection-locked laser networks or degenerate cavity lasers to solve combinatorial optimization problems by finding the ground state of the Ising Hamiltonian [234]. Yao and Zheng also noted the use of nanophotonic systems for simulating quantum-inspired models [220,221]. Quantum photonics is fundamentally required for processing quantum information itself, relevant for future quantum machine learning algorithms operating on quantum data, demanding the same advanced nanophotonic components as QNNs. Zajac et al.’s work on optically controlling adaptive nanoscale domain networks, while classical, illustrates the precision control relevant to quantum systems [239]. Significant challenges persist, including decoherence, high error rates, the scalability of quantum hardware, the development of practical quantum algorithms with proven advantage for AI tasks, and the complex integration of quantum photonics with classical control electronics.

### 5.4. Challenges and Limitations in AI and Computing Applications

Despite the compelling promise and substantial progress, the practical realization and widespread deployment of nanophotonic AI and computing systems face significant, multifaceted challenges demanding sustained research efforts. Integration complexity and scalability constitute a primary hurdle. Most practical systems necessitate hybrid photonic–electronic integration, requiring efficient, high-bandwidth, and low-energy interfaces between photonic computing cores (ONNs and neuromorphic arrays) and electronic control logic, memory, and digital processing. Achieving this through monolithic integration (e.g., silicon photonics with CMOS electronics) or advanced heterogeneous integration techniques presents substantial engineering challenges [203,232,233]. Scaling IPNNs to millions of neurons intensifies issues like waveguide crossing losses, crosstalk, thermal crosstalk between tuning elements (e.g., heaters on MRRs/MZIs), and the physical footprint of components. Strategies like Gu et al.’s MORRs and Qu et al.’s inverse-designed scattering units aim to mitigate footprint limitations [204,205], while Wang et al. and Lian et al. discuss scaling challenges with metasurface integration and photonic memories, respectively [206,240]. Exploiting three-dimensional integration, as in some FSONNs or proposed stacked photonic chips, can enhance density but introduces formidable fabrication and alignment complexities.

Achieving strong, fast, low-energy, and scalable all-optical nonlinearity remains a fundamental bottleneck. While optoelectronic solutions provide a workaround, the inherent latency and energy penalty of O/E/O conversion limit ultimate performance. Material innovations, including novel semiconductors, 2D materials with giant nonlinearities, epsilon-near-zero materials, and optimized PCMs/antimony, and device engineering strategies like resonant enhancement are critical pathways in progress. Programming, calibration, and control present another layer of difficulty. Maintaining precision and stability against thermal drift, fabrication variations, and environmental fluctuations is challenging, particularly for large-scale matrix operations. Techniques like robust training algorithms and on-chip monitoring/feedback are essential, while Sludds et al. note fundamental limits like shot noise [215]. Reconfigurability speed is often hampered by slow tuning mechanisms (e.g., thermal heaters on MRRs); faster electro-optic or all-optical tuning is desirable but difficult. Photonic van der Waals 2D materials offer compact and ultrafast electrical performance [241]. The electronic control overhead for managing thousands or millions of photonic elements can itself become a bottleneck in energy, area, and complexity, potentially offsetting the photonic core’s advantages [214].

Training methodologies adapted to photonic hardware are crucial. In situ training, directly on the physical hardware, is highly desirable but complex due to the difficulty of obtaining gradients through the optical system. Techniques for backpropagation through optical hardware require sophisticated measurement or estimation schemes [205]. Hardware-aware training, run offline but accounting for the specific imperfections, noise, and limitations of the target photonic platform, is vital for achieving robust deployed performance [242,243]. Achieving true system-level energy efficiency requires minimizing all contributors: optical losses (propagation, coupling, and scattering), tuning energy (especially thermal energy), O/E/O penalties in hybrid systems, and peripheral electronics energy (ADCs, DACs, and control logic). Demonstrating end-to-end efficiency surpassing that of optimized electronic ASICs (e.g., TPUs) remains a key goal.

Maximizing the potential of nanophotonics necessitates algorithm–hardware co-design. Developing AI algorithms specifically tailored to leverage photonic strengths (massive parallelism, analog processing, and optical convolutions) while respecting constraints (limited precision, specific nonlinearities, and noise) is essential, rather than merely porting existing digital algorithms. Finally, material and fabrication challenges persist. Reliably developing and fabricating materials with required optical properties (low loss, high nonlinearity, and fast switching), stability, and CMOS compatibility (e.g., GST, Sb, Sc-Sb-Te, novel oxides, and 2D materials) at scale is non-trivial. Fabrication precision and yield for intricate nanophotonic structures (metasurfaces, inverse-designed devices, and dense waveguides) directly impact performance, cost, and commercial viability [244]. Lastly, the huge development of key enabling technologies of optical computing such as electro-optic modulation [245] and photodetection [246] will accelerate commercial deployment.

## 6. Comparative Analysis

Nanophotonics serves as a versatile platform across diverse sectors, yet its specific implementation and physical mechanisms diverge significantly depending on the application domain. In the realm of green energy, the primary objective is broadband efficiency and spectral management [247]. For instance, perovskite solar cells utilize nanostructured light-trapping schemes to maximize absorption across the solar spectrum, pushing efficiencies beyond 25.5%. This contrasts with CSP and STPV, where the focus shifts to spectral selectivity, such as engineering surfaces that exhibit high absorptance for incoming sunlight but low thermal emittance to retain heat at temperatures exceeding 1000 °C. While energy applications prioritize broad spectral harvesting and thermal retention, biosensing and computing applications rely heavily on precise resonance and wave propagation [248]. Biosensors exploit high-sensitivity phenomena like LSPR and SERS to detect minute refractive index changes or molecular fingerprints, achieving single-molecule sensitivity that energy-harvesting devices do not require [8,33,249]. Similarly, optical computing leverages the wave nature of light, not for absorption but for performing linear transformations and matrix multiplications through diffraction and interference, necessitating low-loss propagation rather than confinement.

Nanophotonics has improved medicine and healthcare: non-invasive diagnostics and therapeutics are facilitated by the use of nanophotonic materials like black phosphorous and gold nanoparticles, which offer tunable optical properties for precise targeting and treatment. These materials are used in photodynamic therapy, bioimaging, and multimodal imaging, providing new avenues for effective cancer treatment [33,250,251]. Material selection is another critical point of divergence driven by functional requirements and environmental constraints. Green energy technologies increasingly favor perovskites for their tunable bandgaps and refractory metals like tungsten and molybdenum for their stability under extreme thermal stress in CSP and STPV systems. In contrast, the medical and biosensing fields predominantly utilize plasmonic noble metals (gold and silver) and carbon-based nanomaterials due to their biocompatibility and ability to generate localized heat for therapies or enhance signals for detection. However, these materials face distinct stability challenges; while perovskites struggle with moisture and UV degradation, silver-based sensors are susceptible to oxidation, requiring protective dielectric coatings [240,252,253]. The computing sector diverges further by prioritizing CMOS-compatible materials like silicon and phase-change materials (e.g., GST) to facilitate integration with electronic logic and non-volatile memory, a constraint that does not apply to the other fields [254,255,256]. Overall, the reciprocal development between artificial neural networks and nanophotonics is driving innovations in optical computing, enabling new functionalities and enhancing performance in various applications (Table 2).

The scalability and fabrication hurdles also vary sharply across these domains. Solar energy applications demand cost-effective, large-area manufacturing techniques, such as roll-to-roll processing or solution processing for perovskites, to maintain economic viability. This stands in opposition to the high-precision, often costly fabrication methods required for optical computing and advanced biosensors, such as electron-beam lithography, which are necessary to define the intricate nanostructures for SERS substrates or metasurfaces but currently limit mass production. Furthermore, while energy systems grapple with macro-scale efficiency and long-term durability, healthcare applications face the unique challenge of operating within complex biological matrices, where biofouling and matrix interference can severely compromise sensor performance, necessitating advanced surface functionalization that is irrelevant to solar or computing hardware.

## 7. Conclusions and Outlook

This review has synthesized recent breakthroughs in nanophotonics, underscoring its pivotal role in advancing green energy, precision medicine, biosensing, and optical computing. Nanophotonic engineering enables unprecedented control over light at subwavelength scales, driving innovations such as perovskite solar cells with >30% efficiency, plasmonic biosensors capable of single-molecule detection, and tumor-selective photothermal therapies. In computing, integrated photonic neural networks and metasurface-based platforms offer transformative gains in speed and energy efficiency, potentially overcoming the fundamental limitations of electronic systems. Despite these advances, key hurdles impede large-scale commercialization. Material stability remains critical, particularly for perovskite photovoltaics under operational stresses and plasmonic nanostructures susceptible to oxidation. Fabrication complexity and cost hinder scalability; techniques like e-beam lithography for SERS substrates or cleanroom-dependent photonic circuits are economically prohibitive for mass production. System integration, especially the coupling of nanophotonic components with electronic control units, demands sophisticated heterogenous packaging, increasing design overhead. Additionally, the standardization of performance metrics and regulatory frameworks for biomedical applications is underdeveloped. The commercialization of nanophotonic technology depends on addressing existing challenges through material innovation. This can be achieved by first developing robust, eco-friendly nanomaterials (e.g., lead-free perovskites and oxidation-resistant plasmonic alloys) and hybrid systems (e.g., MOF-encapsulated nanoparticles) that balance performance with stability and then merging these materials with manufacturing advancements such as scaling low-cost techniques like nanoimprint lithography for metasurfaces, roll-to-roll processing for solar cells, and 3D printing for biosensors to supplant expensive high-precision methods. Concurrently, AI co-design must leverage machine learning for inverse nanostructure design, fabrication optimization, and real-time sensor analytics to enhance performance while curtailing prototyping costs. Multidisciplinary integration is equally vital, converging photonics, electronics, and biotechnology to develop “lab-on-chip” diagnostics and energy-efficient photonic accelerators compatible with existing infrastructure. Finally, establishing standardized testing protocols and incentivizing public–private partnerships through supportive regulatory and economic models will de-risk scaling pilot technologies like nanophotonic thermophotovoltaics and implantable biosensors. As these efforts mature, coordinated research in scalable manufacturing and cross-sector collaboration will transition nanophotonics from laboratory breakthroughs toward enabling sustainable energy grids, accessible diagnostics, and ultra-efficient computing systems.

The authors would like to acknowledge and thank the Egypt Scholars organization and especially Advanced Labs for organizing the teamwork required to produce this paper.

## Figures and Tables

**Table 2:** Summary of nanophotonic applications.

Area	Application	Nanophotonic Mechanism	Primary Material	Primary Challenge	Reference
Solar Energy	Perovskite photovoltaics	Light trapping	Perovskites TiO_2_	Stability UV degradation	[59,60,61,63,64,65,69,70,75,78,257]
	Concentrated solar power	High absorption Low emission	Refractory metals	Thermal stability at high temperatures	[85,86,87,89,90,258]
	Thermophotovoltaics	Thermal emission control	Tungsten	Wavelength spectral matching	[95,96,100,101,102,103,259]
Biosensing	Biomolecule detection	LSPR, SERS, refractive index sensing	Plasmonic metals	Fabrication cost	[8,33,249]
	Environmental monitoring	Fluorescence	Quantum dots	Emission specificity in different media	[110,112]
	Food safety	Autofluorescence	Organic materials	Emission specificity in different media	[129,130]
Medicine	Photothermal therapy	Light-to-heat conversion	Gold nanoparticles	Penetration depth	[143,144,145,146,148,149,150]
	Chemotherapy	Light-to-heat conversion	Gold nanoparticles	Penetration depth	[185,186,187,188,189]
	Image-guided surgery	NIR fluorescence	Quantum dots	Tissue penetration	[190,191,192]
Healthcare	Tumor diagnostics	Light absorption	Plasmonic metals	Stability and fabrication cost	[112,165,166,167]
	Imaging modality	Material reflection	Organic materials	Penetration depth	[190,193]
	mRNA detection	Light absorption	Plasmonic metals	Stability and fabrication cost	[176,178,179,182,183,184]
Optical Computing	Optical neural networks	Diffraction Phase modulation	Silicon metasurface	Scalability issue	[203,219,220,221,240,252,253]
	Neuromorphic computing	Resistive switching	Phase-change materials	Integration issue	[227,228,236,254,255,256]

## Data Availability

No new data were created or analyzed in this study. Data sharing is not applicable to this article.
